# Supplementary figures and images for: Barriers to cervical cancer prevention and triage strategies: a study of knowledge, attitudes, and p16/Ki-67 dual-staining utility among high-risk women in Tuoli and Fuyun counties, Xinjiang
Source: PeerJ. 2025 Oct 2;13:e20100. doi: 10.7717/peerj.20100 (PMC12497396; doi:10.7717/peerj.20100)

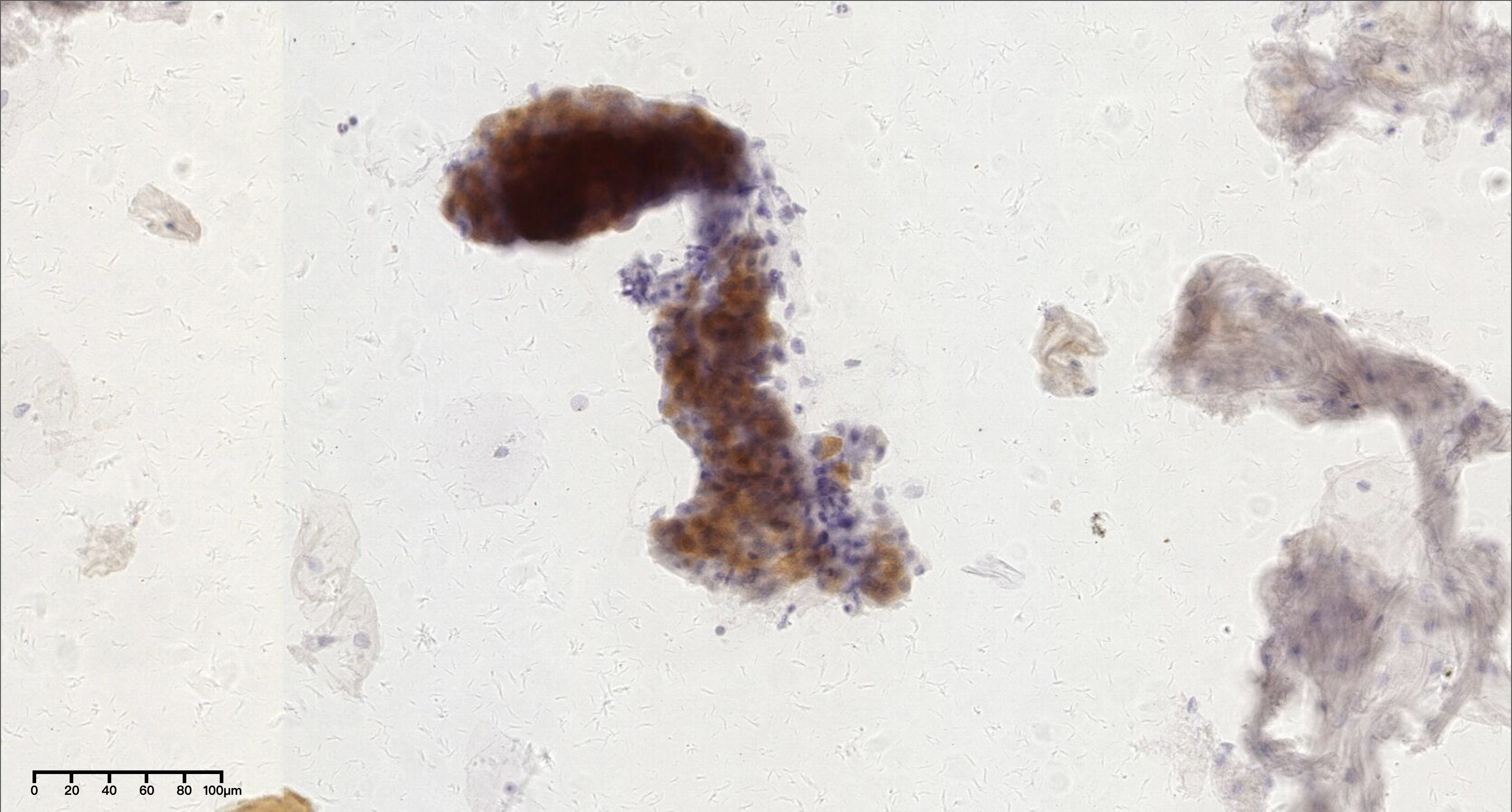

Supplement: Supplemental Information 1 — Brownish-yellow stained cervical epithelial cells that were considered positive for p16. [file peerj-13-20100-s001.png]

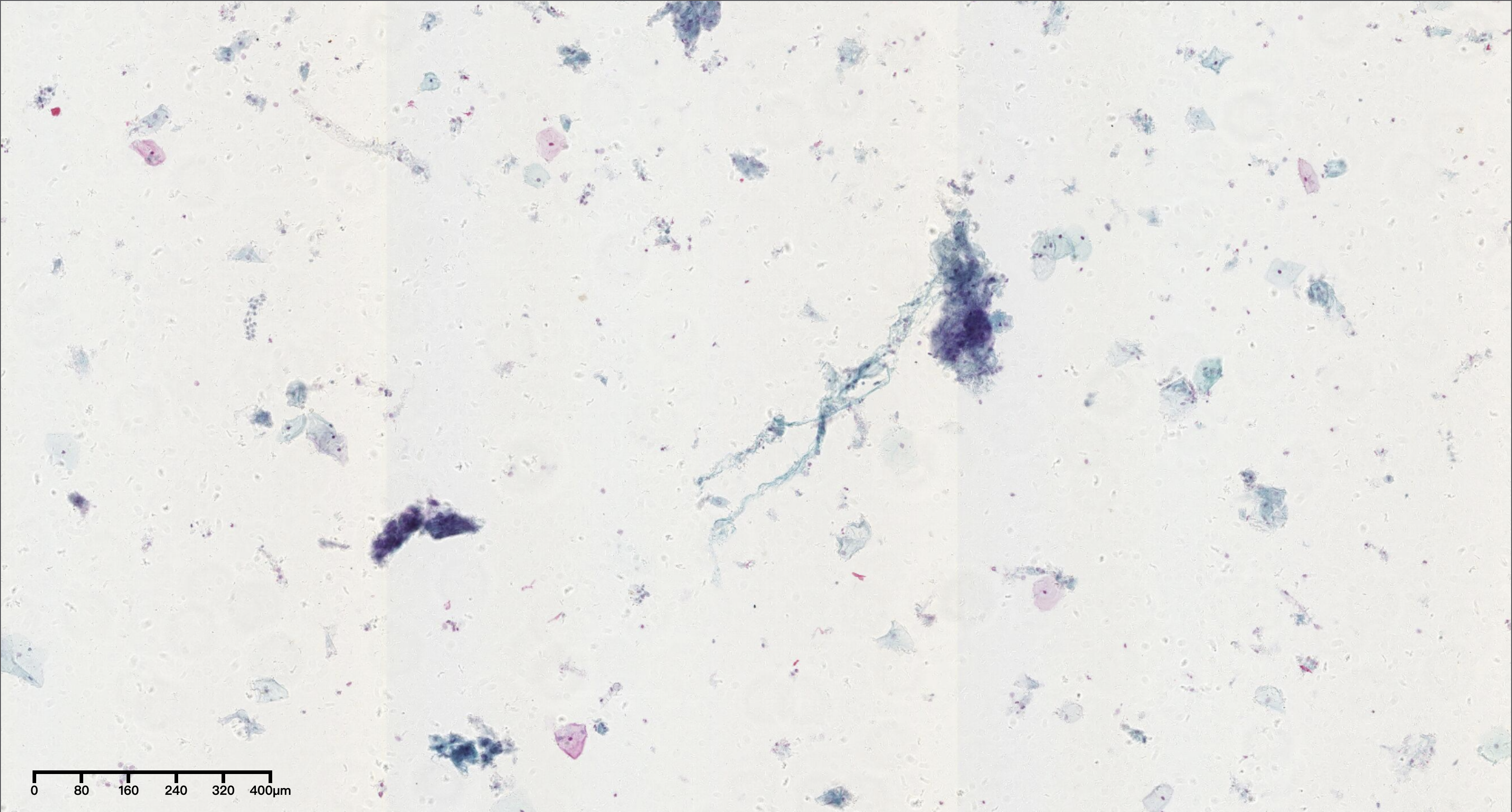

Supplement: Supplemental Information 2 — Staining images (HSIL, high-grade squamous intraepithelial lesion) [file peerj-13-20100-s002.png]

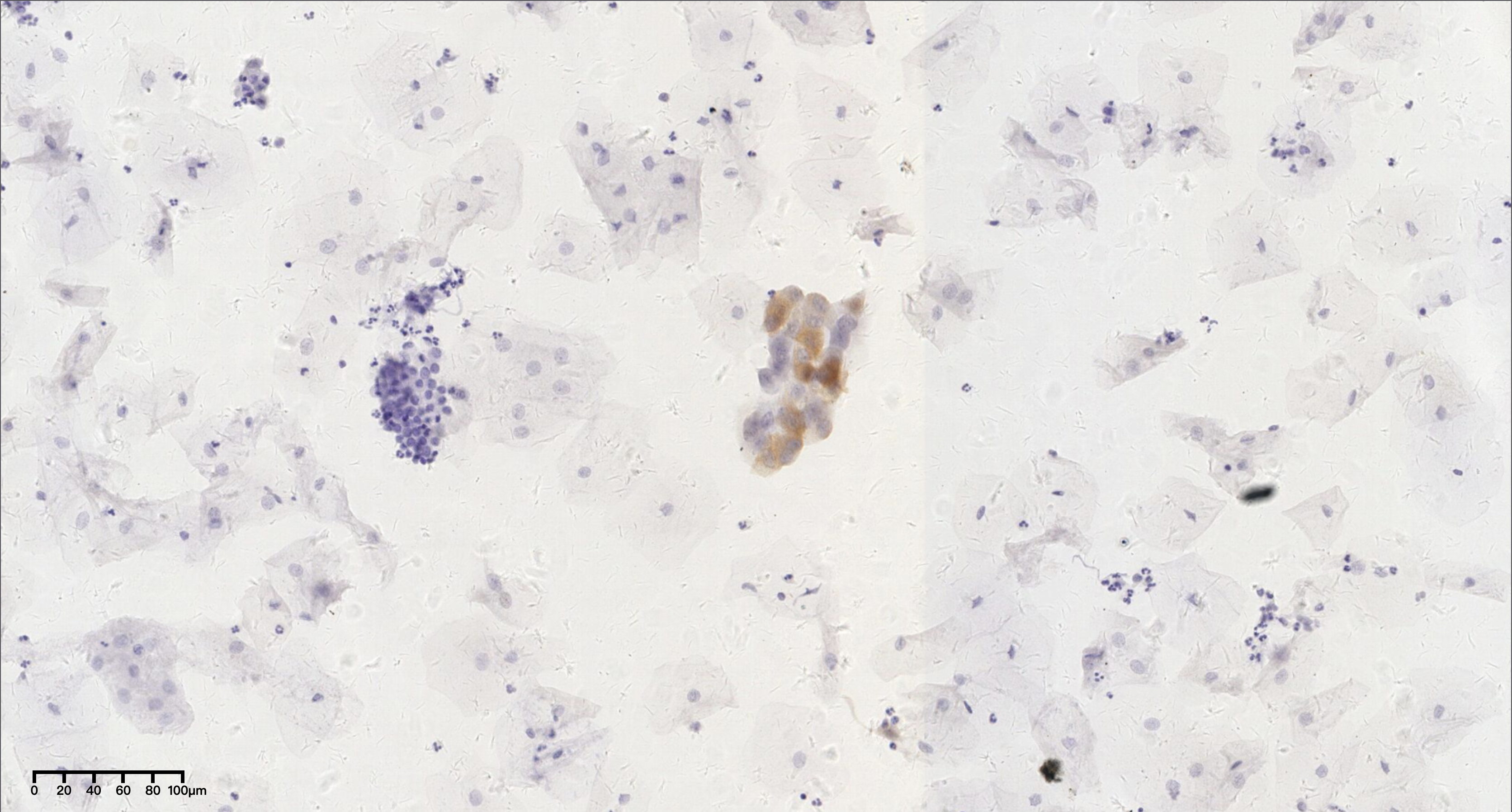

Supplement: Supplemental Information 3 — Brownish-yellow stained cervical epithelial cells that were considered positive for p16. [file peerj-13-20100-s003.png]

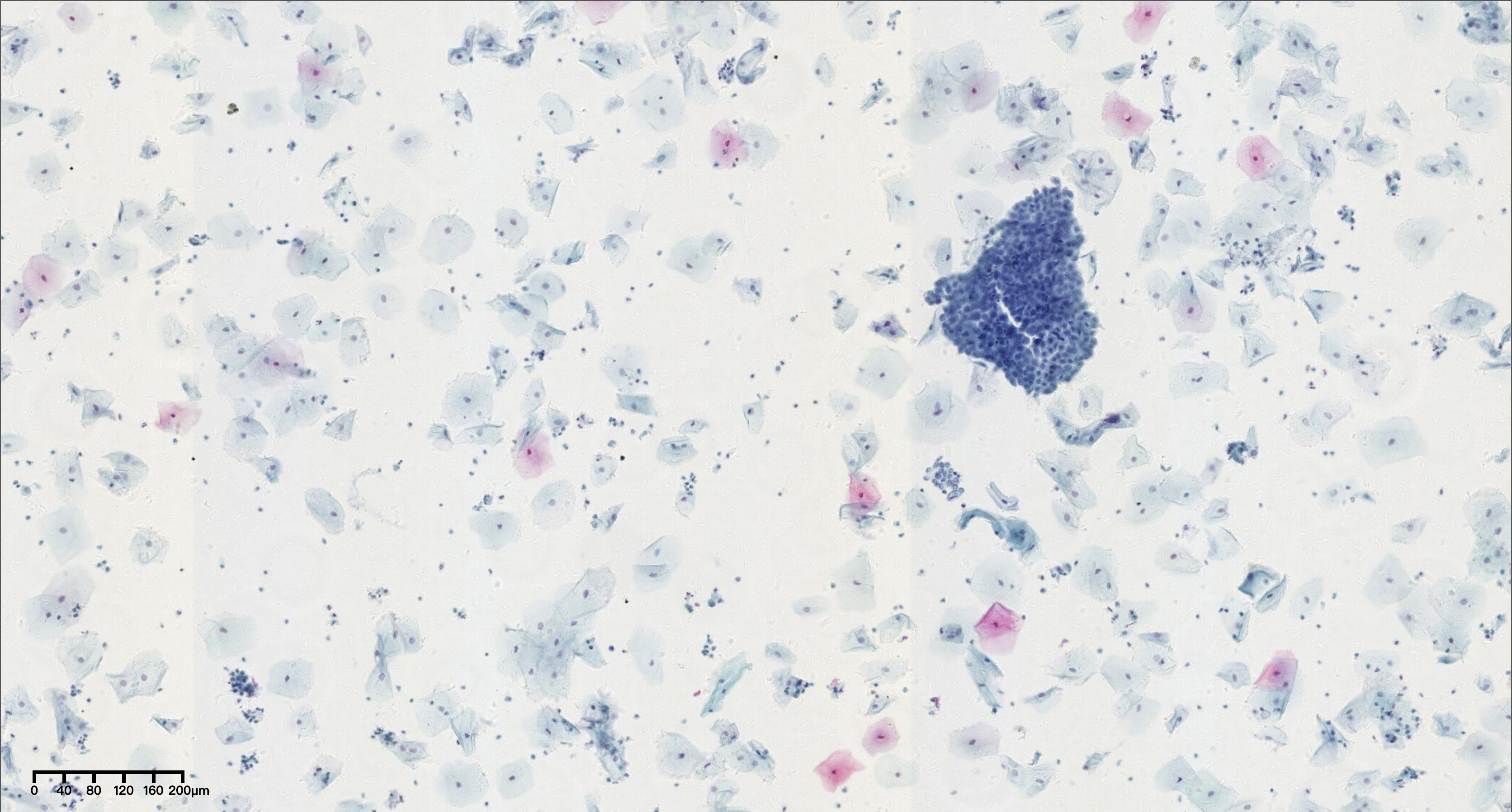

Supplement: Supplemental Information 4 — Staining images (LSIL, low-grade squamous intraepithelial lesion) [file peerj-13-20100-s004.png]

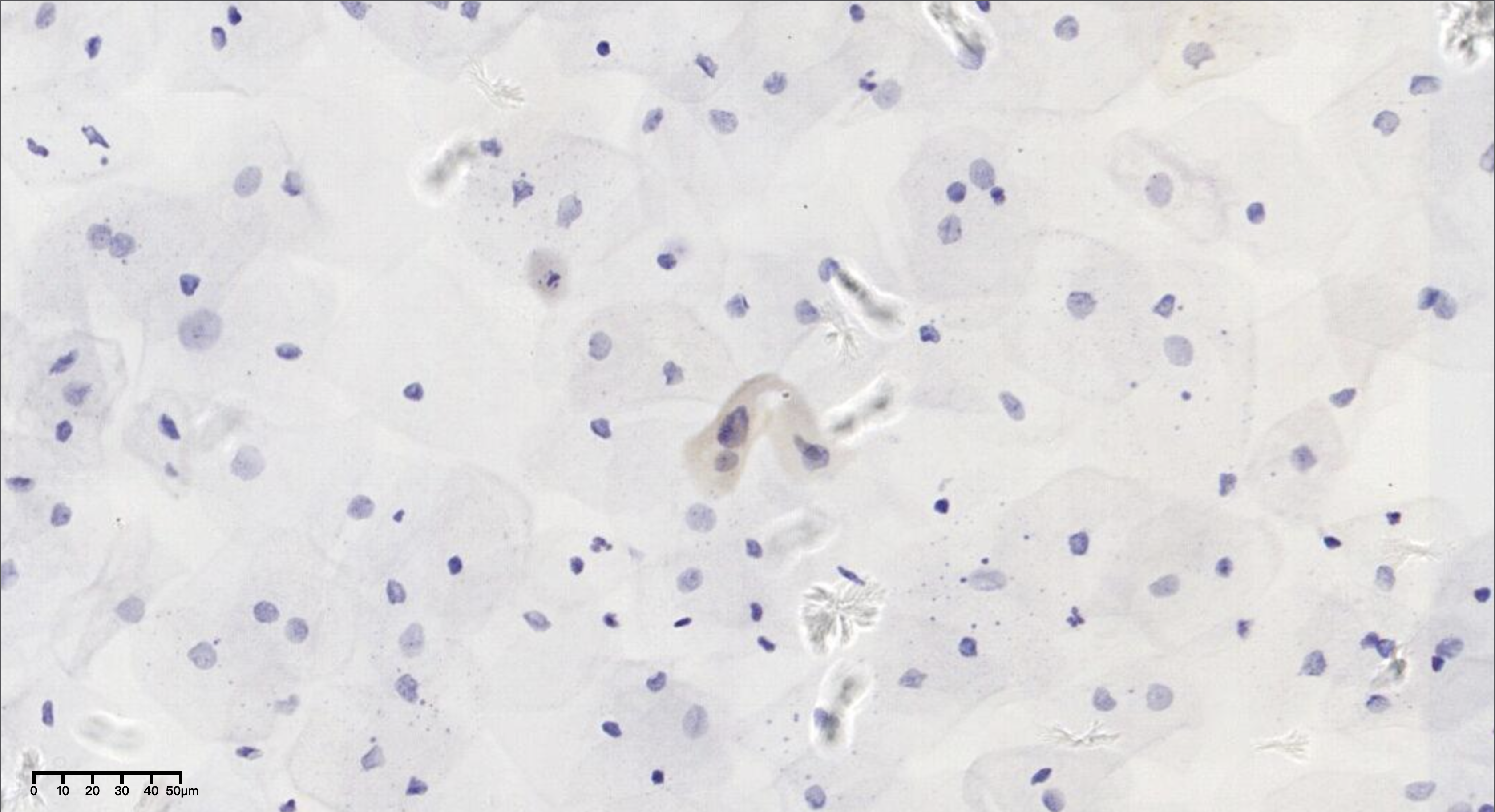

Supplement: Supplemental Information 5 — Brownish-yellow stained cervical epithelial cells that were considered positive for p16. [file peerj-13-20100-s005.png]

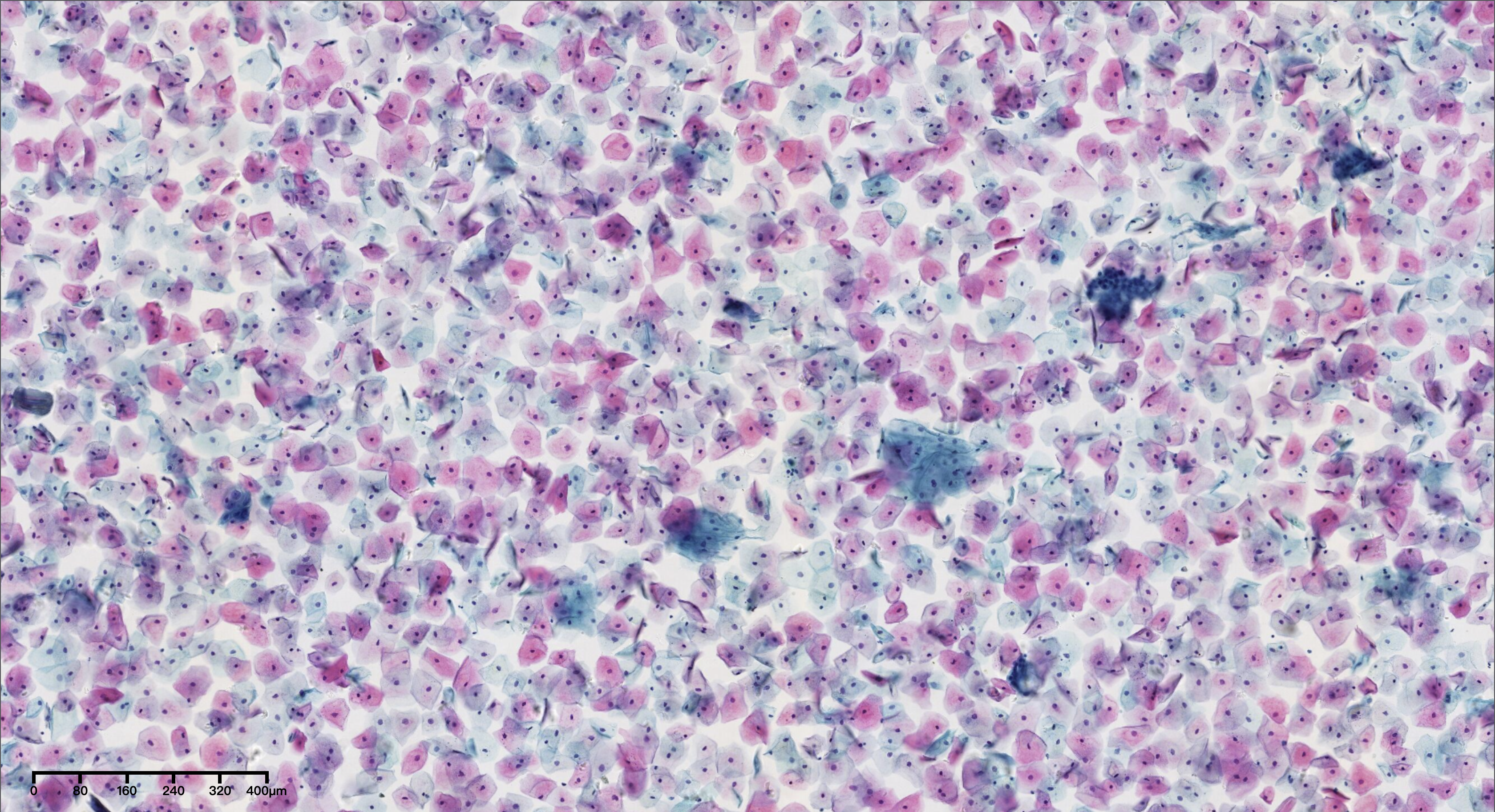

Supplement: Supplemental Information 6 — Staining images (LSIL, low-grade squamous intraepithelial lesion) [file peerj-13-20100-s006.png]

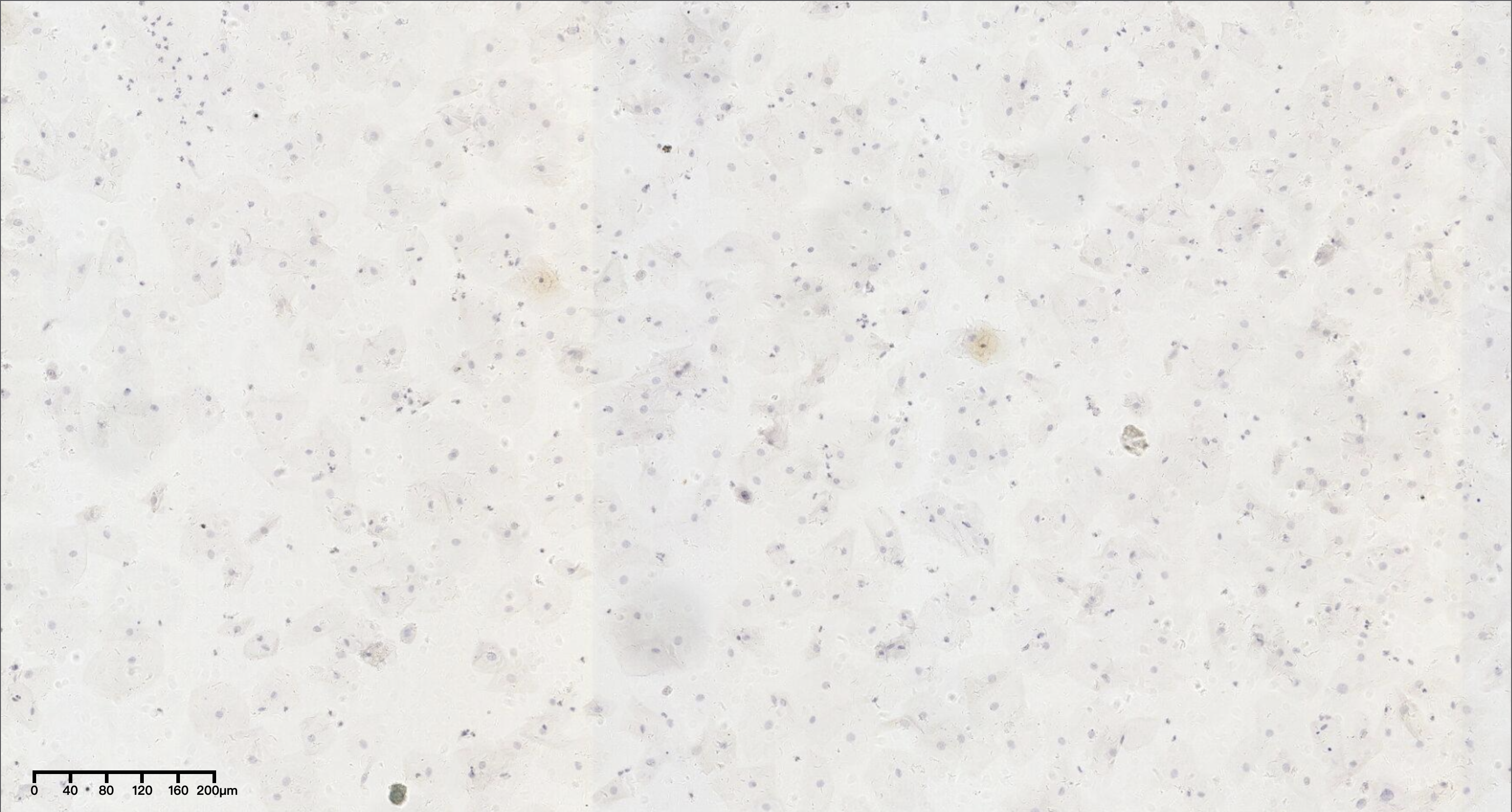

Supplement: Supplemental Information 7 — Brownish-yellow stained cervical epithelial cells that were considered positive for p16. [file peerj-13-20100-s007.png]

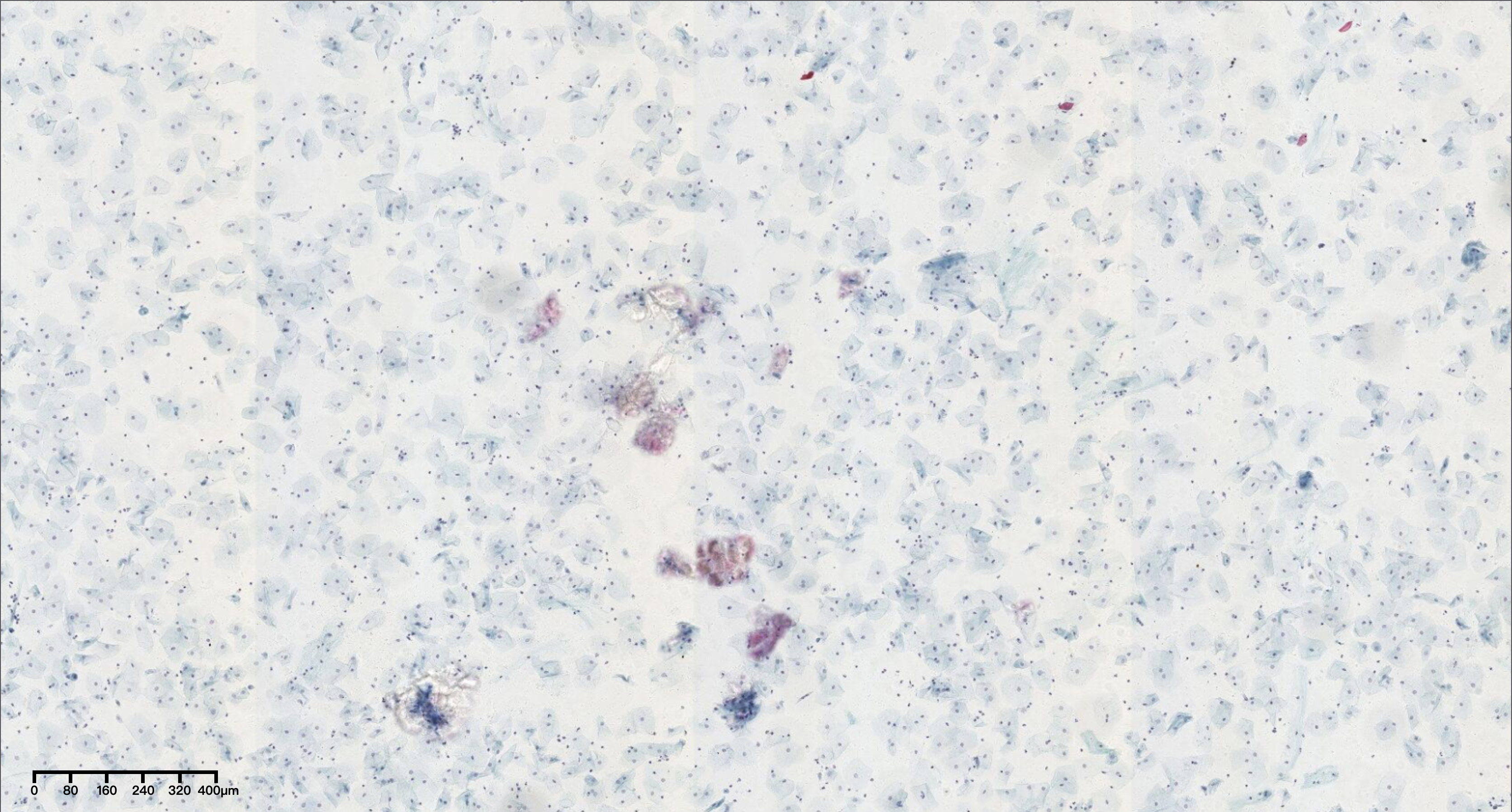

Supplement: Supplemental Information 8 — Staining images (LSIL, low-grade squamous intraepithelial lesion) [file peerj-13-20100-s008.png]

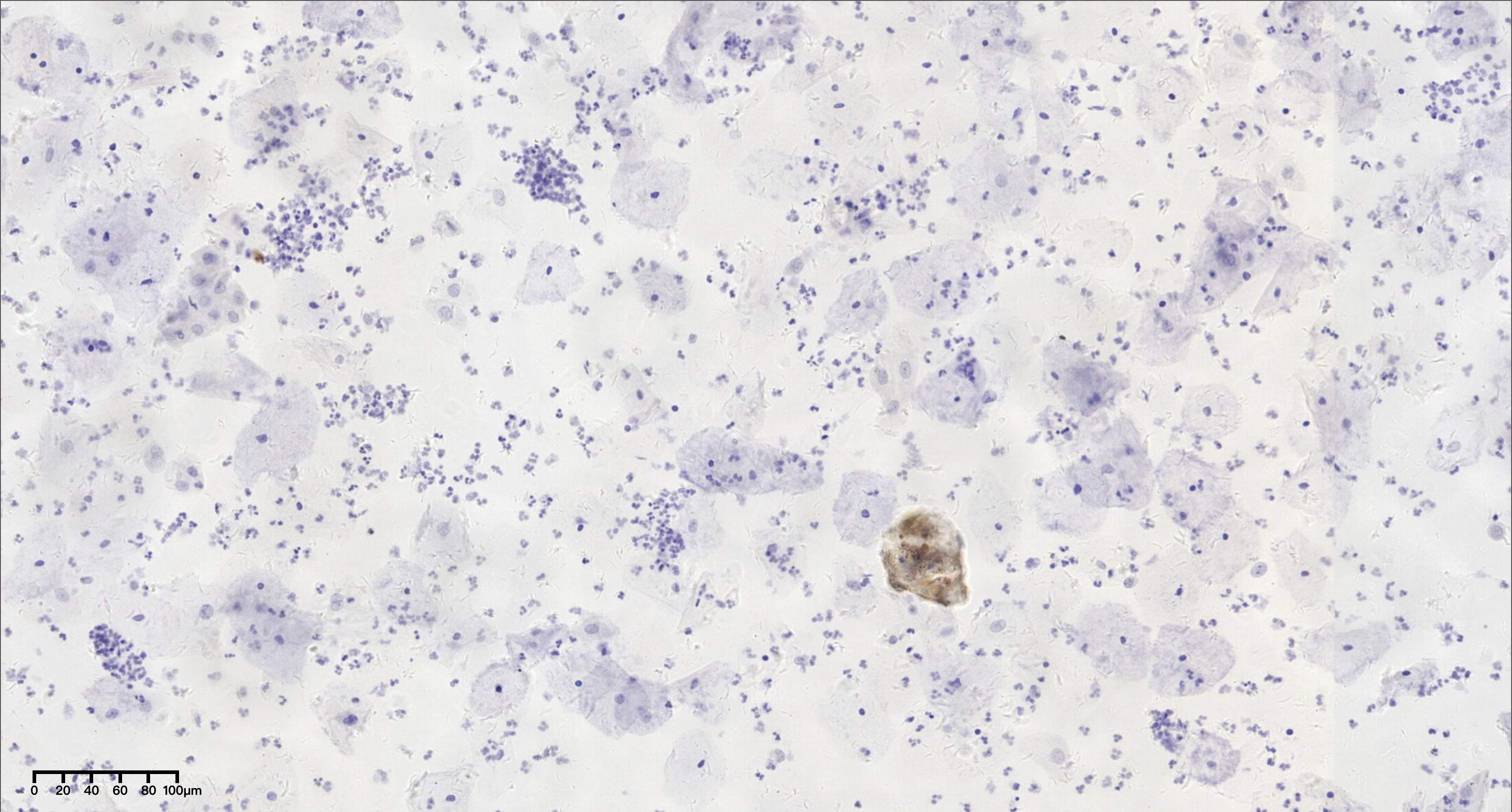

Supplement: Supplemental Information 9 — Brownish-yellow stained cervical epithelial cells that were considered positive for p16. [file peerj-13-20100-s009.png]

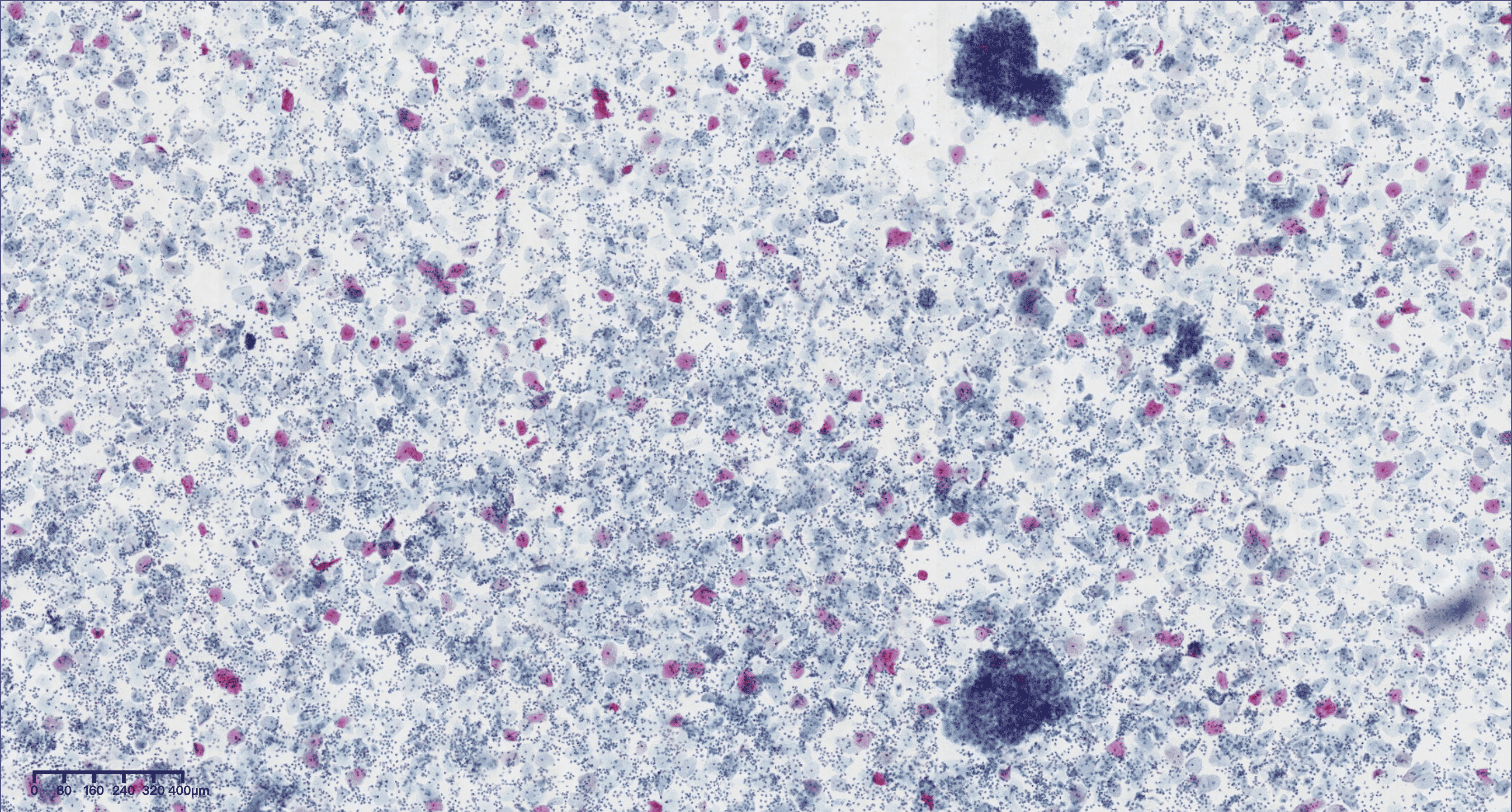

Supplement: Supplemental Information 10 — Staining images (LSIL, low-grade squamous intraepithelial lesion) [file peerj-13-20100-s010.png]

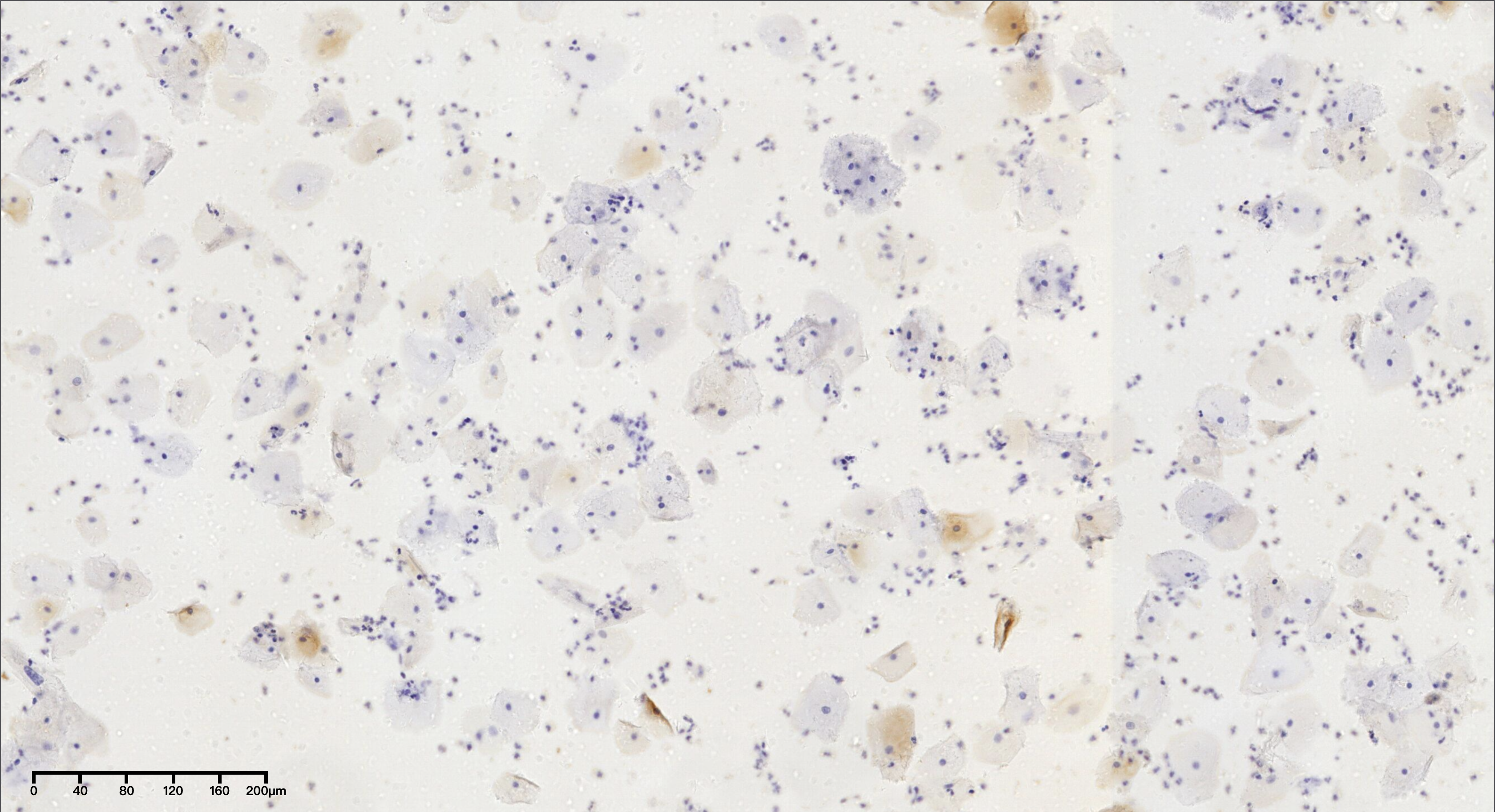

Supplement: Supplemental Information 11 — Brownish-yellow stained cervical epithelial cells that considered positive for p16. [file peerj-13-20100-s011.png]

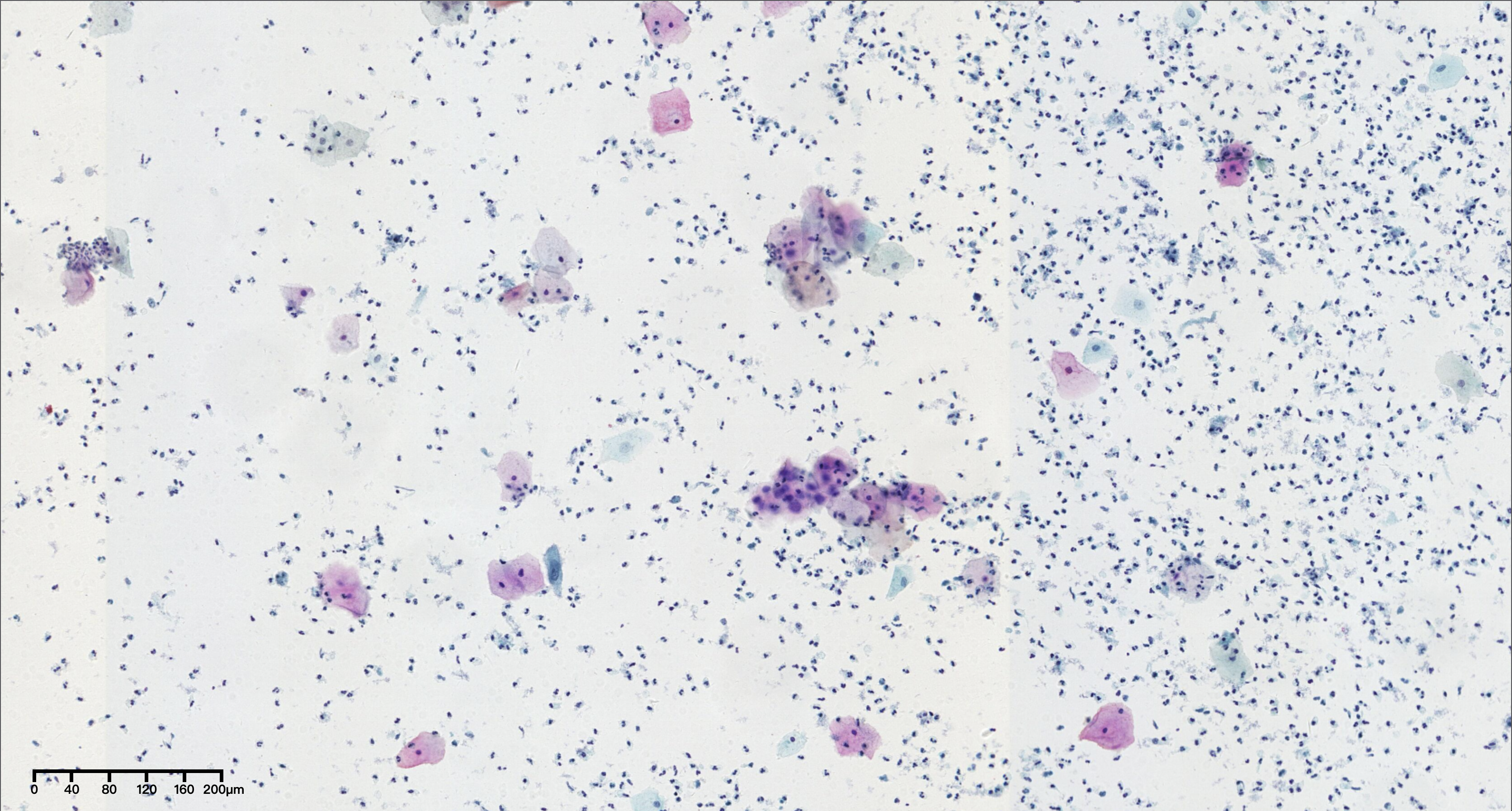

Supplement: Supplemental Information 12 — Staining images (LSIL, low-grade squamous intraepithelial lesion) [file peerj-13-20100-s012.png]

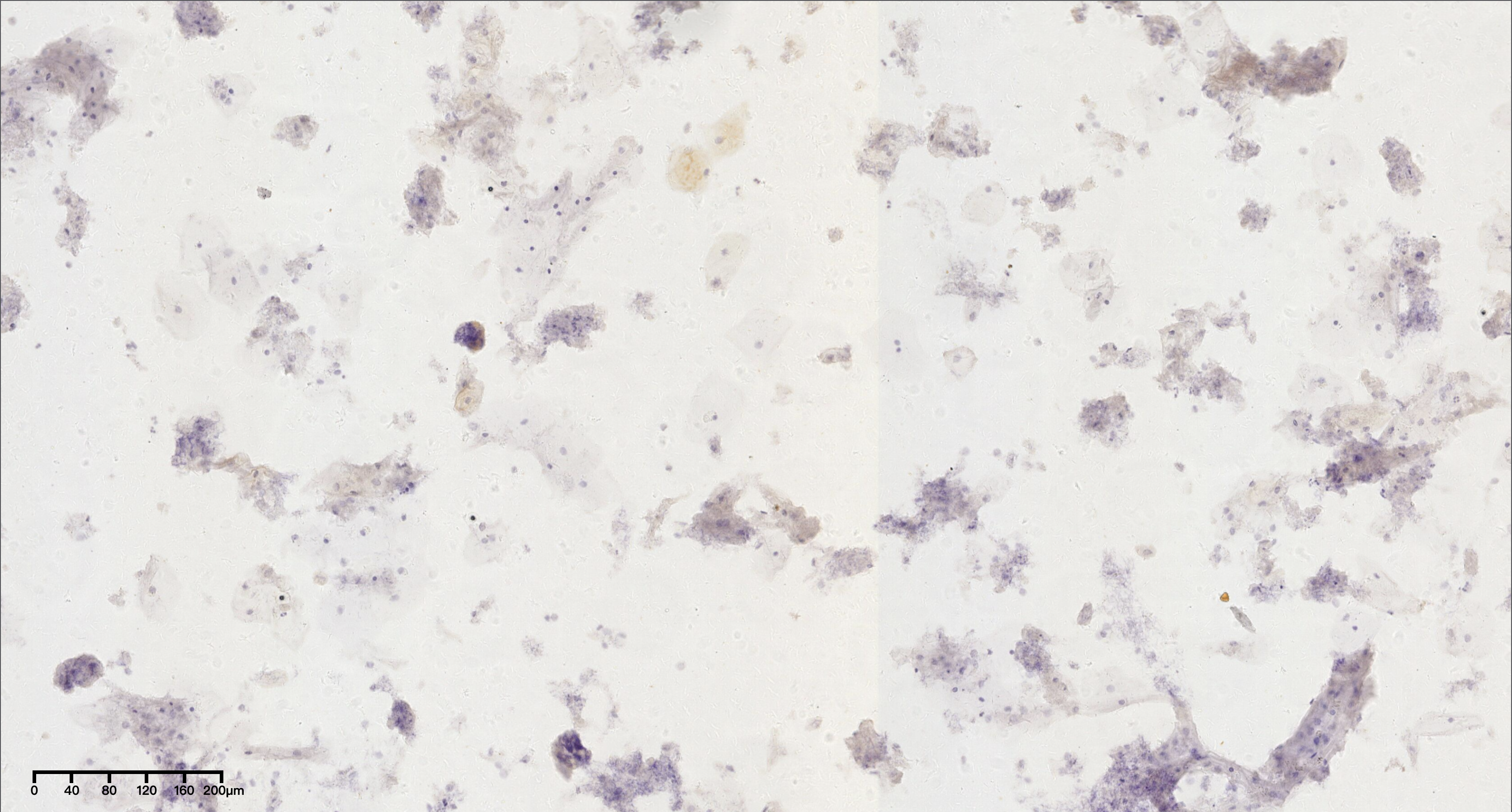

Supplement: Supplemental Information 13 — Brownish-yellow stained cervical epithelial cells that were considered positive for p16. [file peerj-13-20100-s013.png]

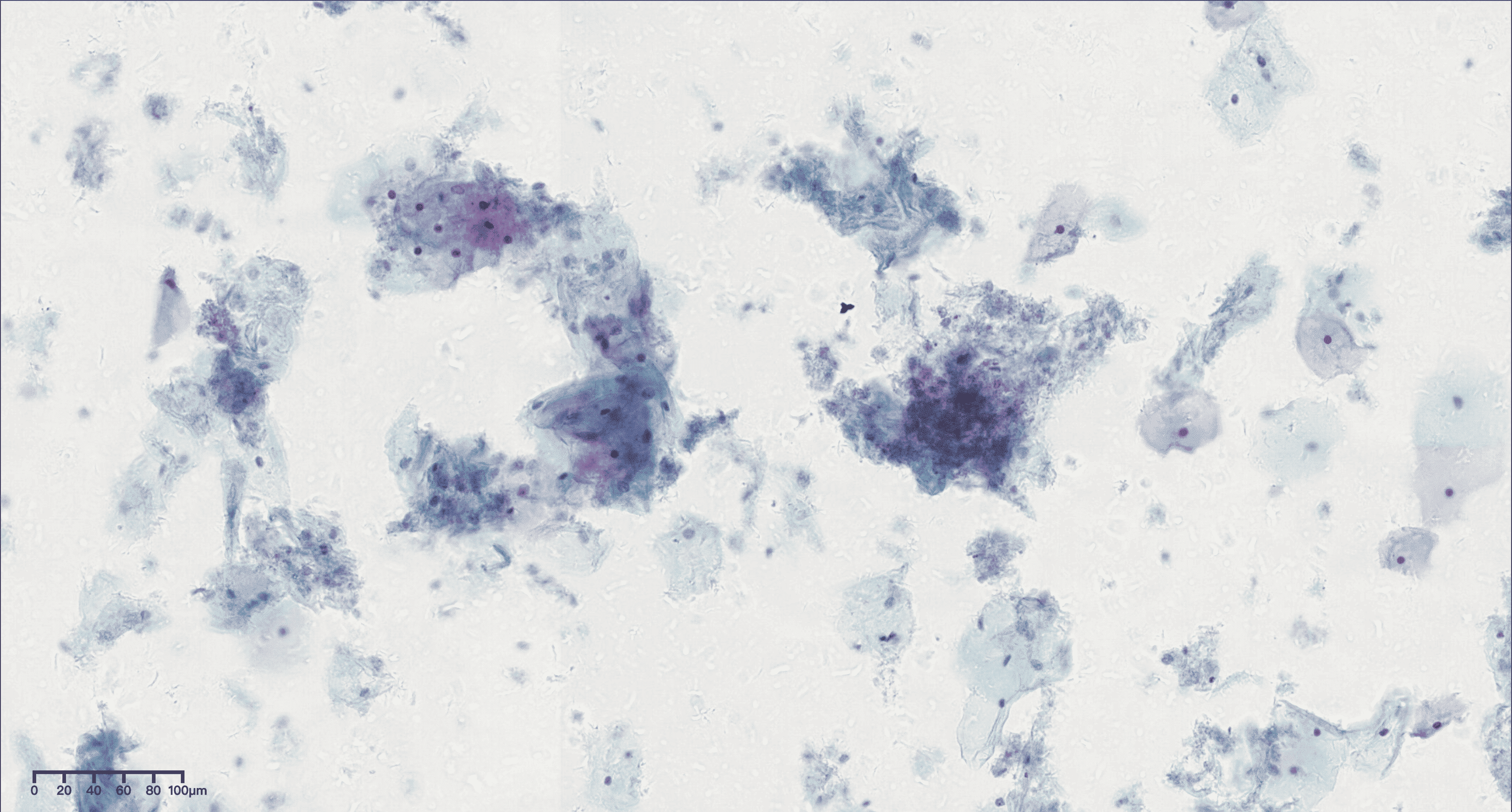

Supplement: Supplemental Information 14 — Staining images (LSIL, low-grade squamous intraepithelial lesion) [file peerj-13-20100-s014.png]

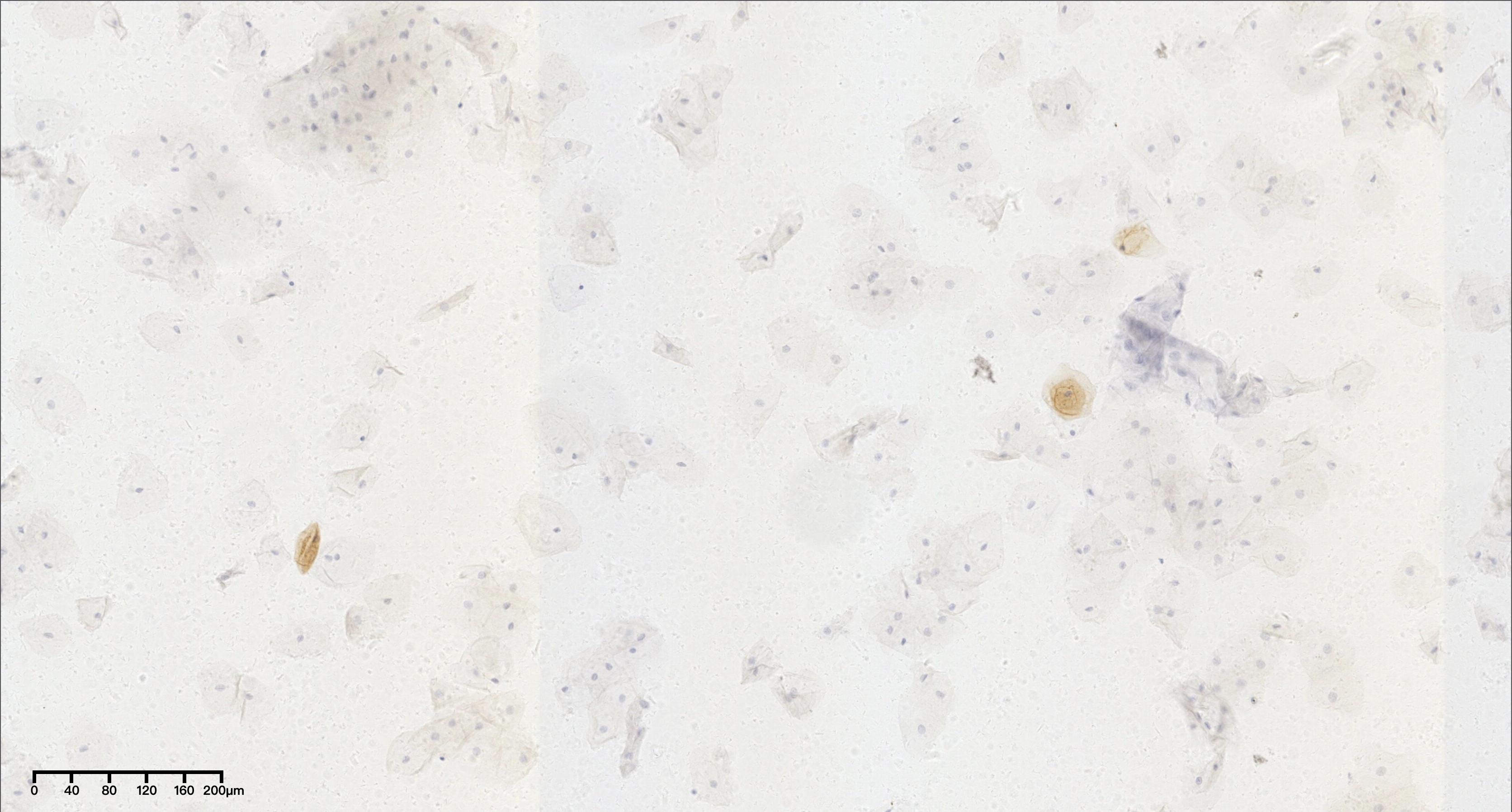

Supplement: Supplemental Information 15 — Brownish-yellow stained cervical epithelial cells that were considered positive for p16. [file peerj-13-20100-s015.png]

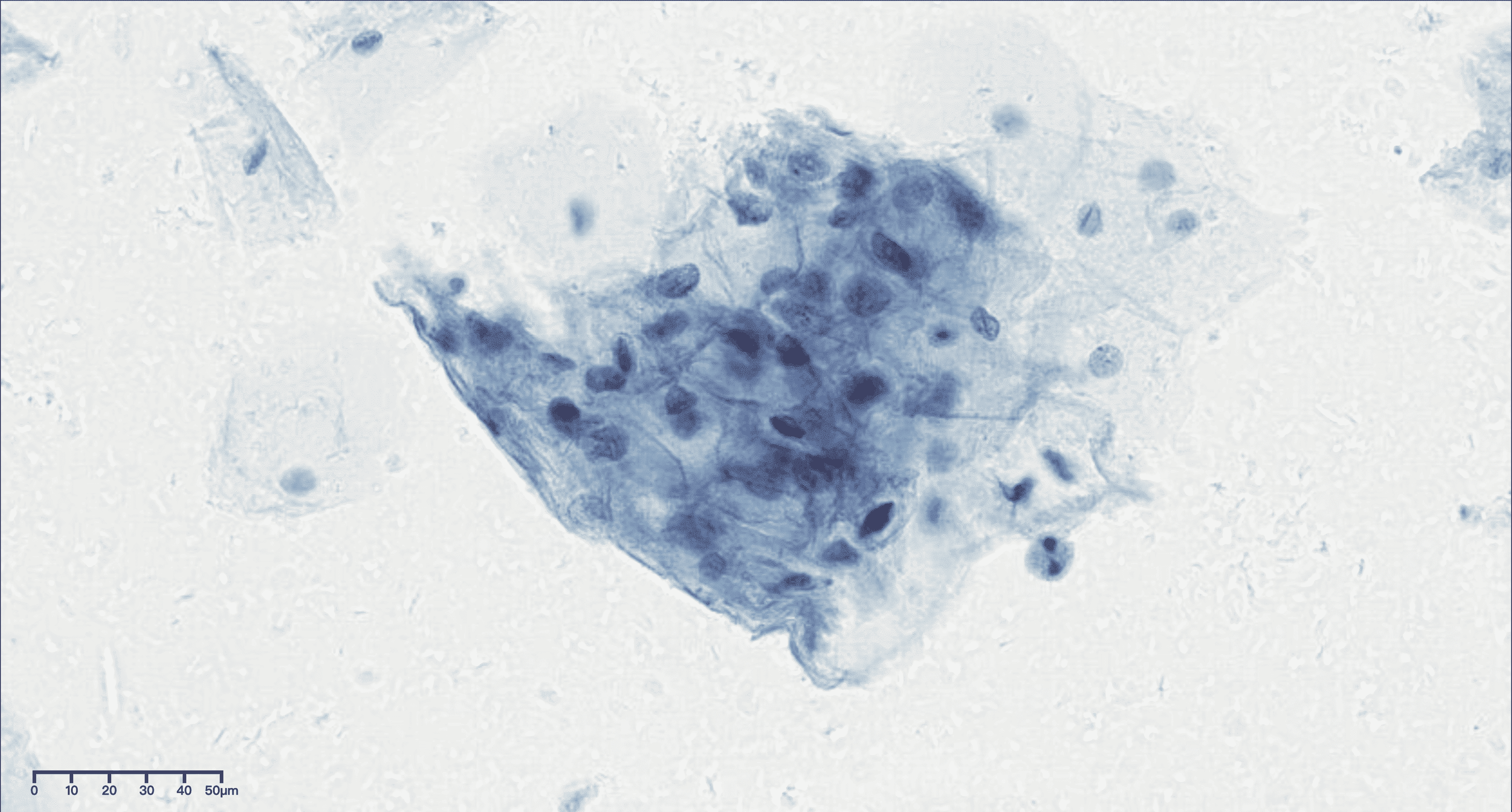

Supplement: Supplemental Information 16 — Staining images (LSIL, low-grade squamous intraepithelial lesion) [file peerj-13-20100-s016.png]

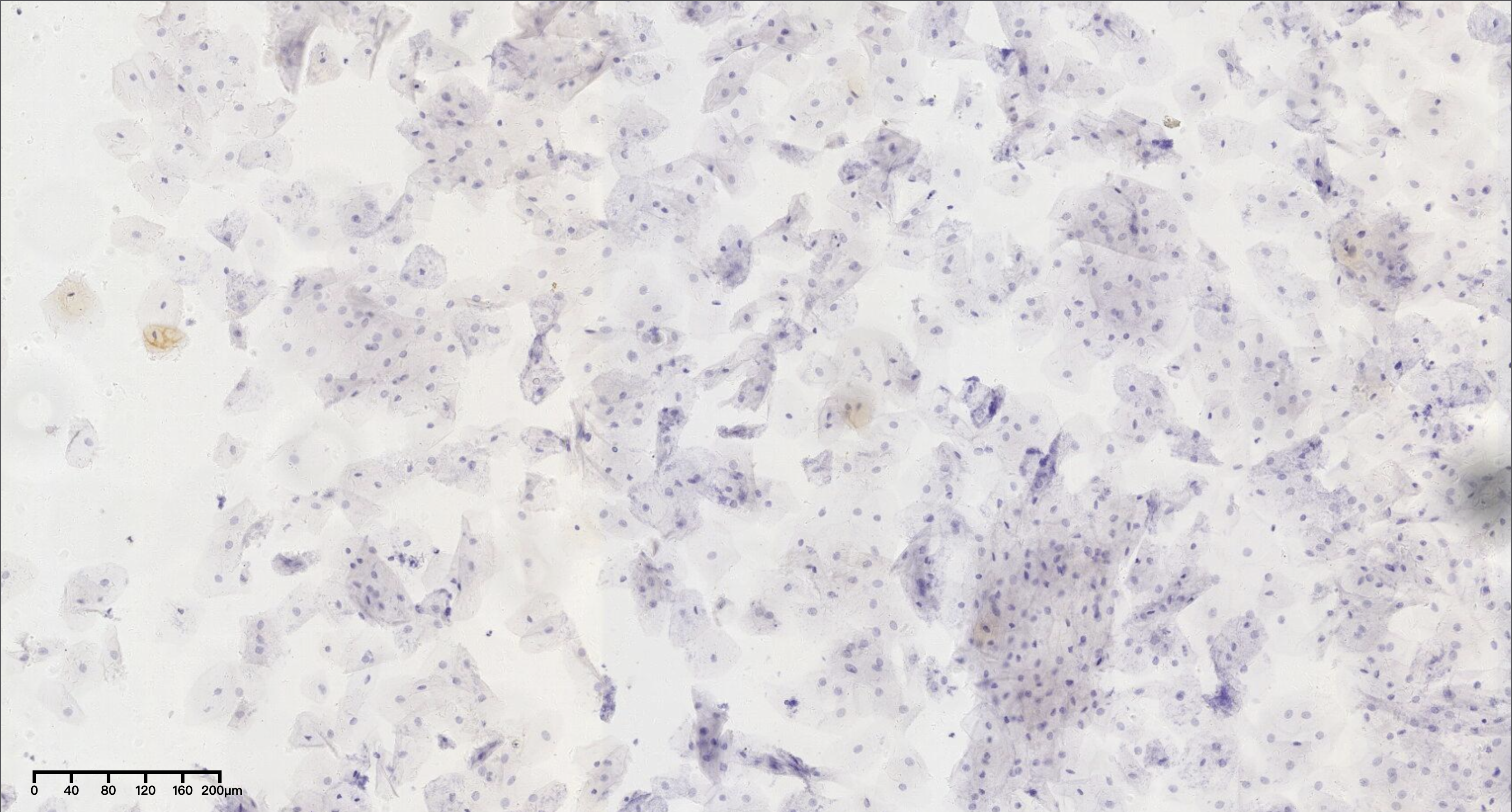

Supplement: Supplemental Information 17 — Brownish-yellow stained cervical epithelial cells that considered positive for p16. [file peerj-13-20100-s017.png]

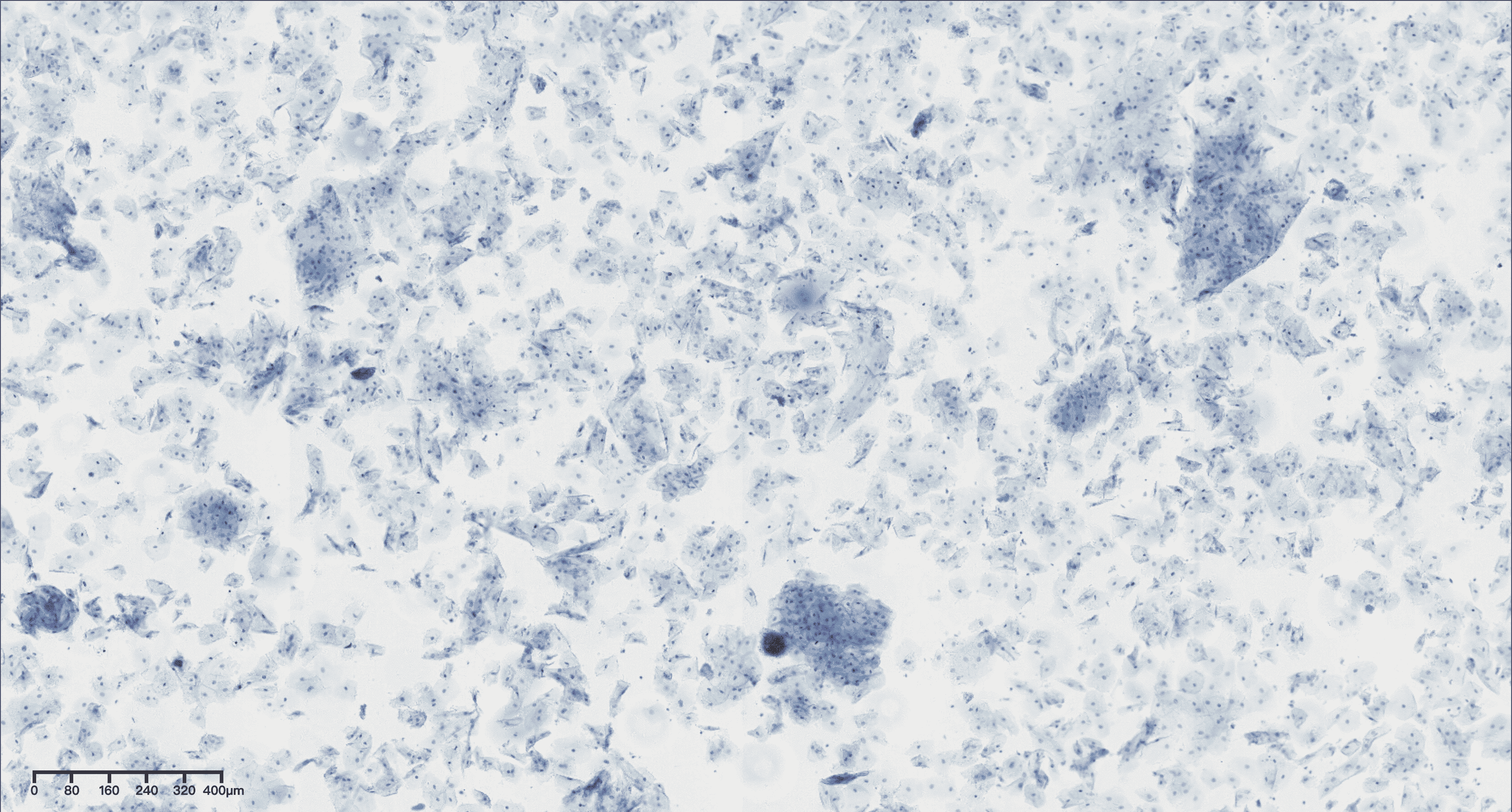

Supplement: Supplemental Information 18 — Staining images (LSIL, low-grade squamous intraepithelial lesion) [file peerj-13-20100-s018.png]

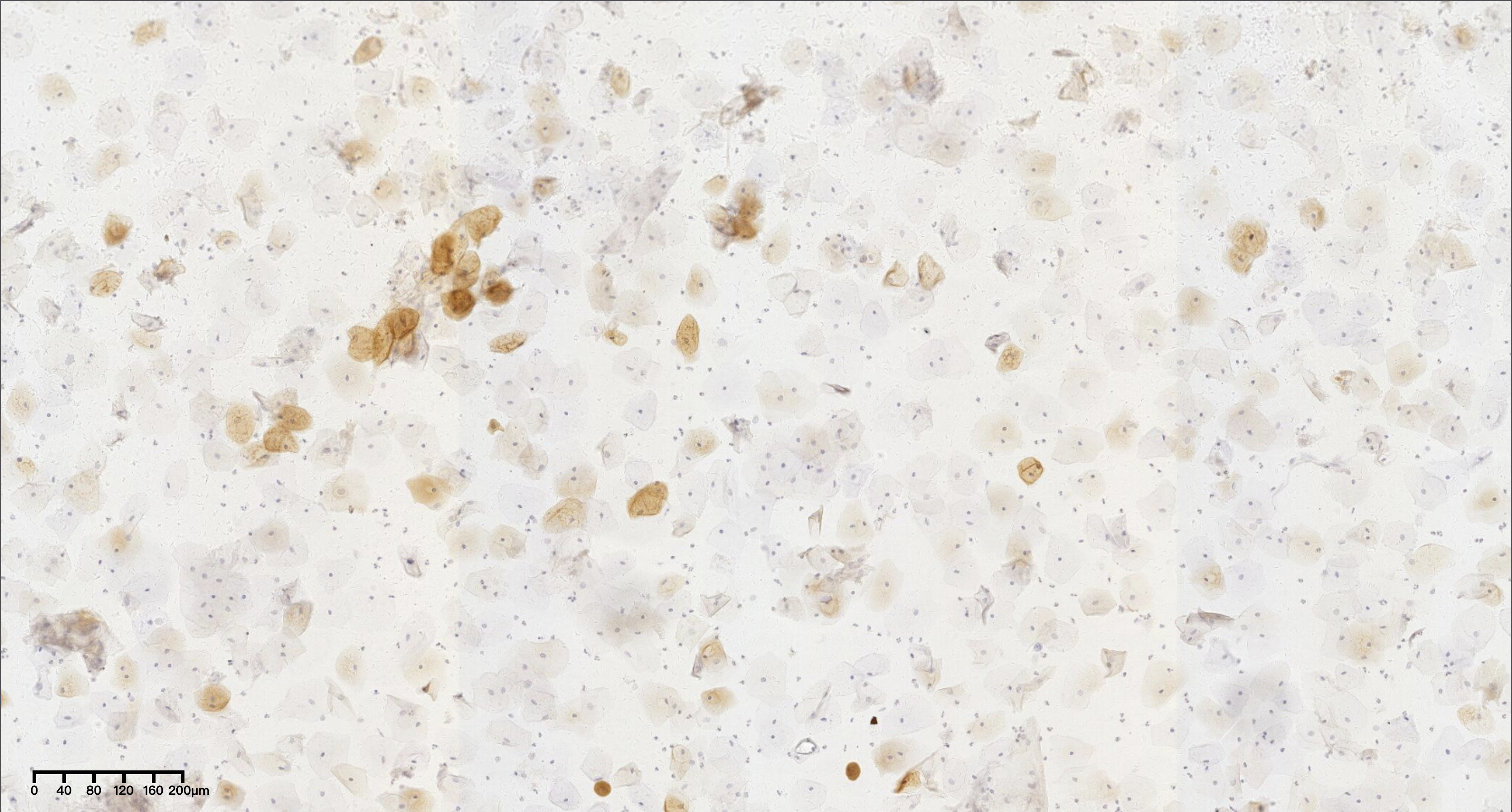

Supplement: Supplemental Information 19 — Brownish-yellow stained cervical epithelial cells that considered positive for p16. [file peerj-13-20100-s019.png]

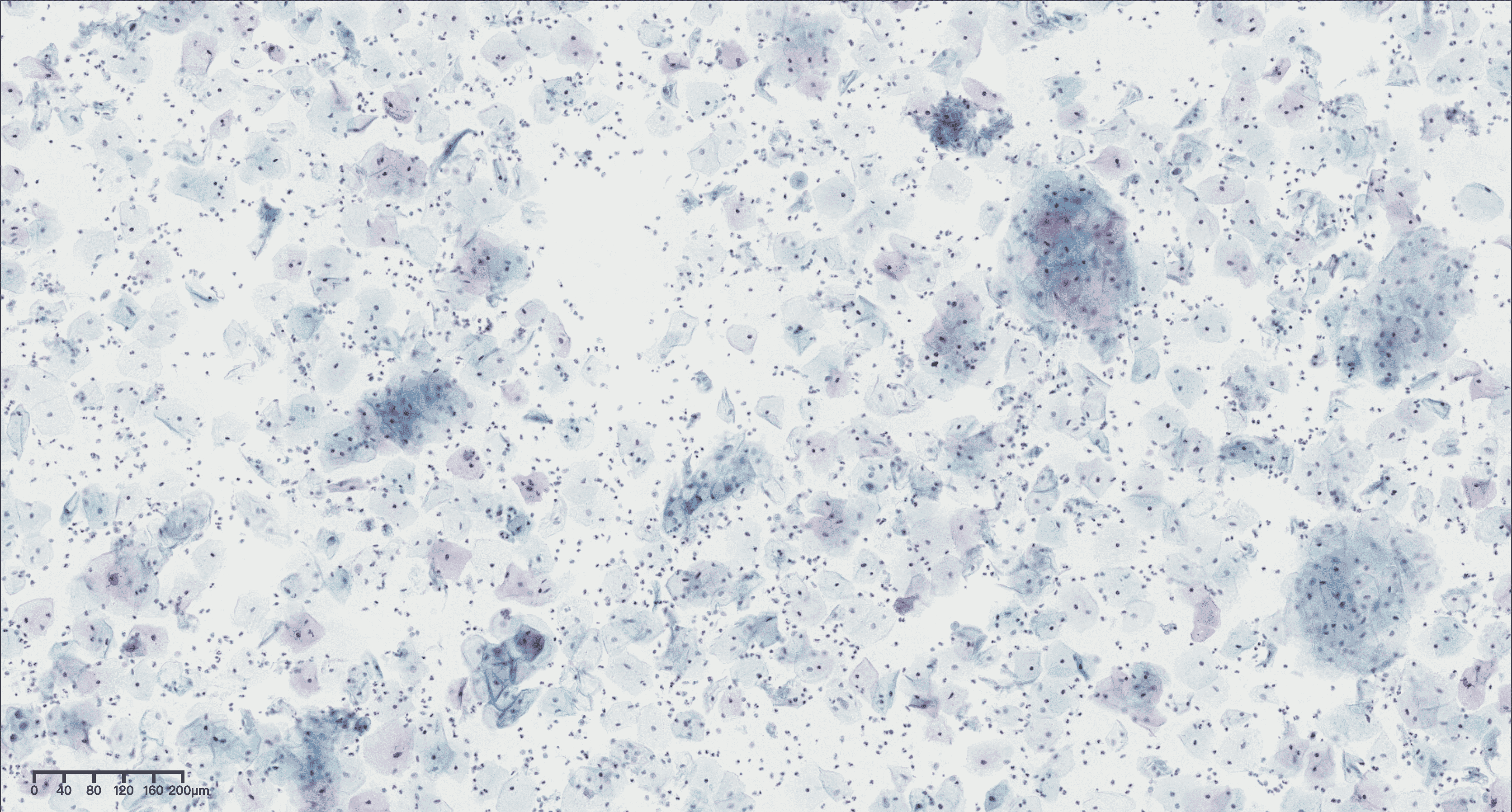

Supplement: Supplemental Information 20 — Staining images (LSIL, low-grade squamous intraepithelial lesion) [file peerj-13-20100-s020.png]

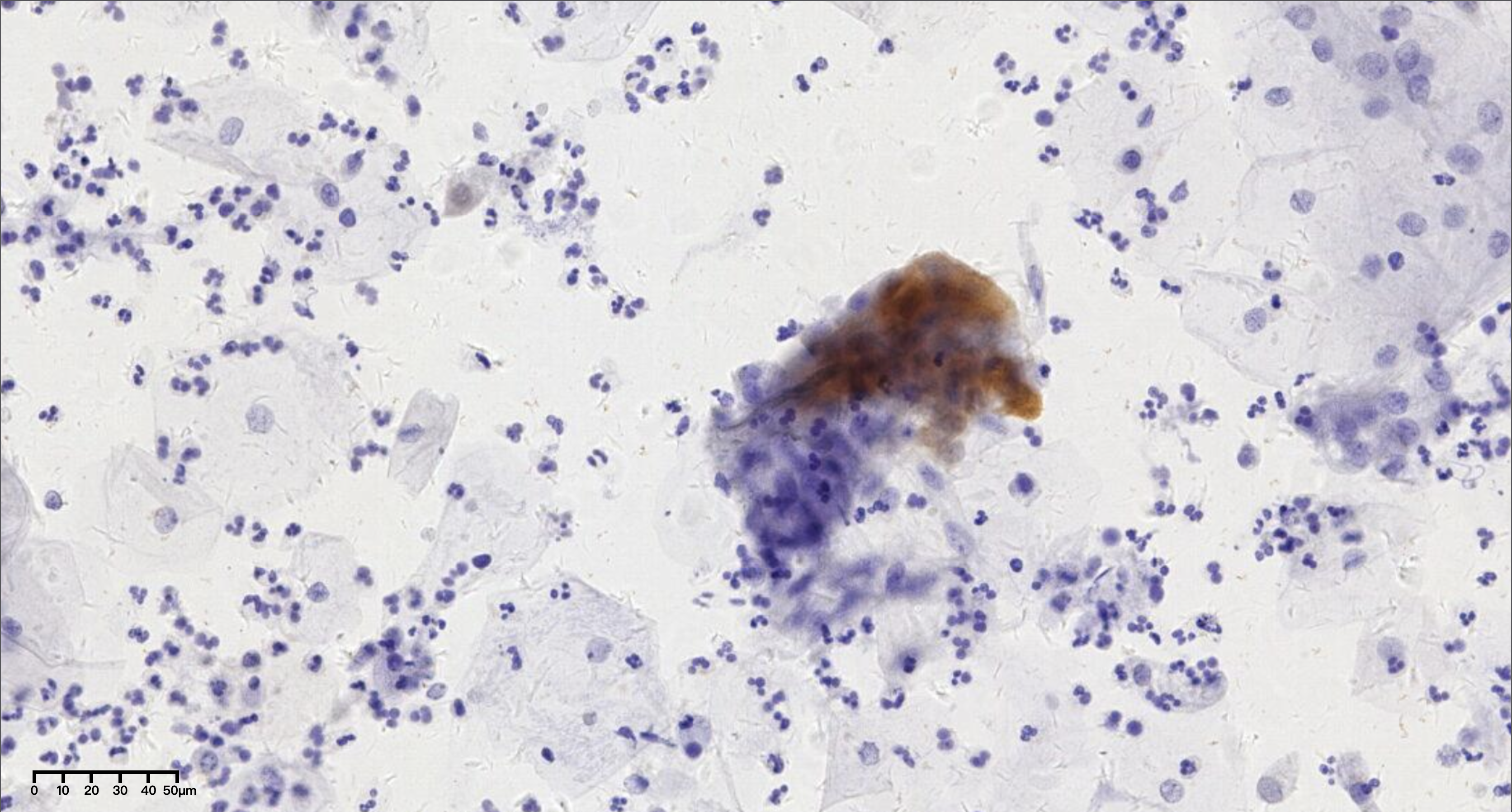

Supplement: Supplemental Information 21 — Brownish-yellow stained cervical epithelial cells that considered positive for p16. [file peerj-13-20100-s021.png]

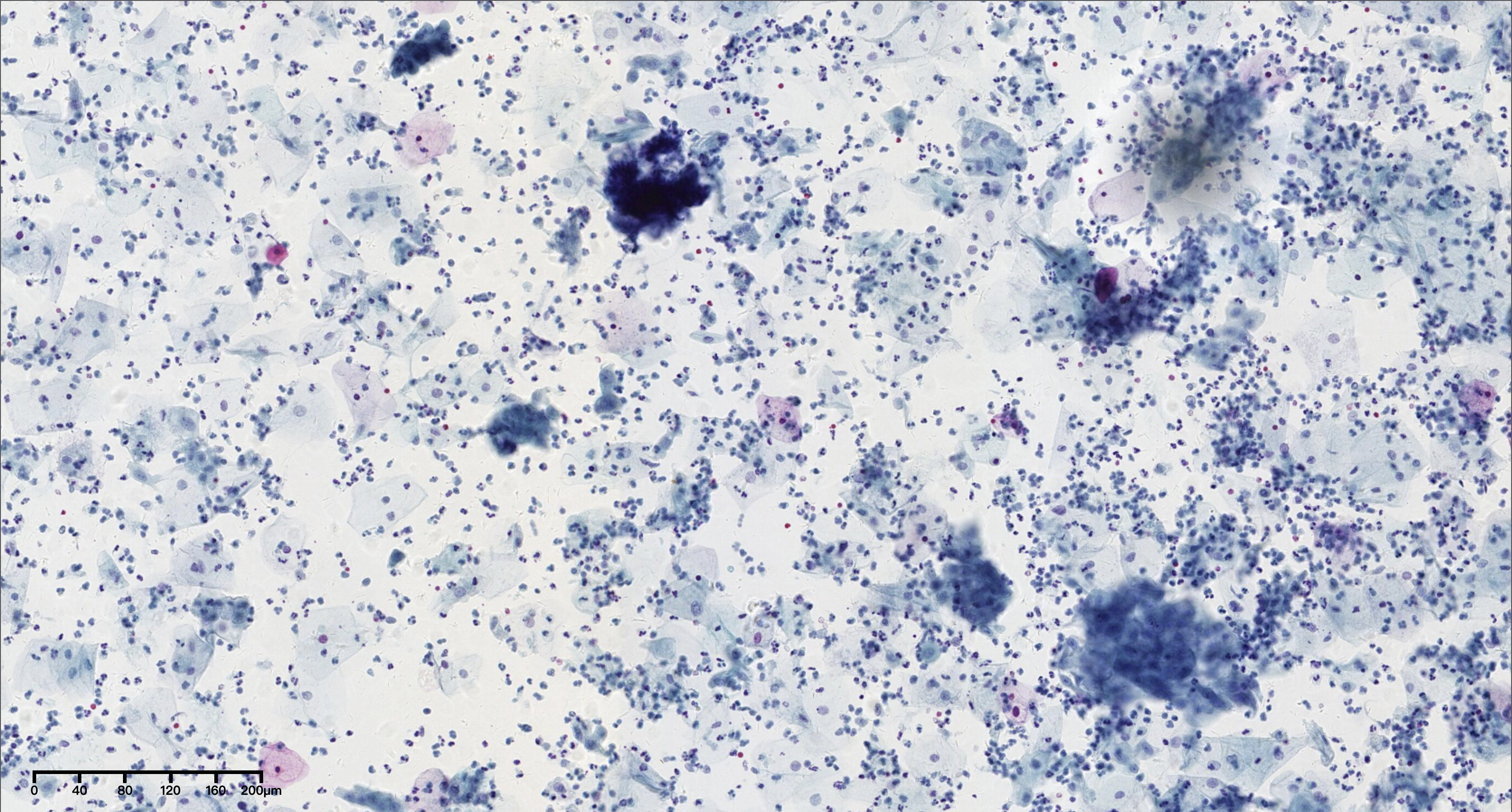

Supplement: Supplemental Information 22 — Staining images (LSIL, low-grade squamous intraepithelial lesion) [file peerj-13-20100-s022.png]

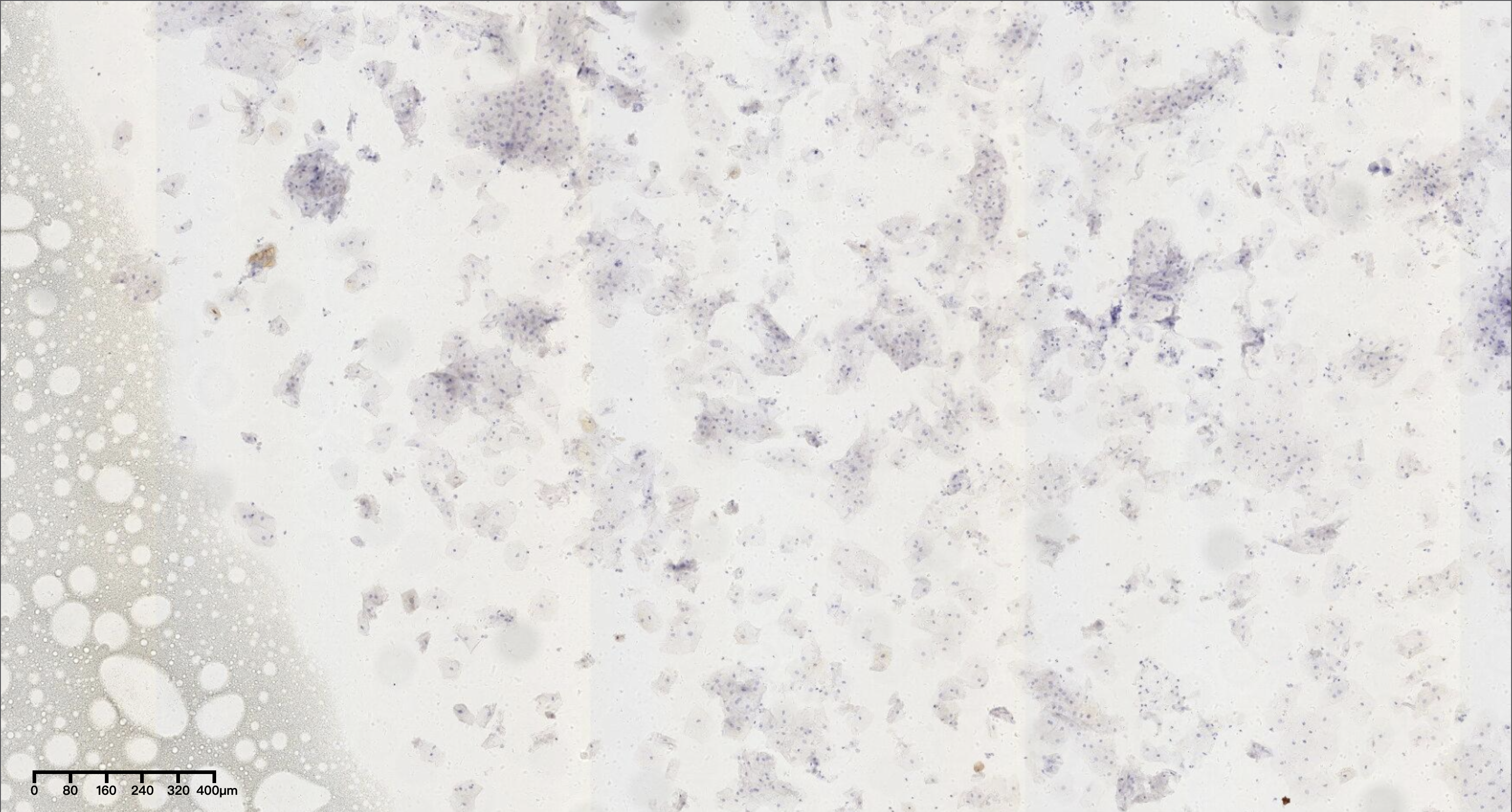

Supplement: Supplemental Information 23 — Brownish-yellow stained cervical epithelial cells that considered positive for p16. [file peerj-13-20100-s023.png]

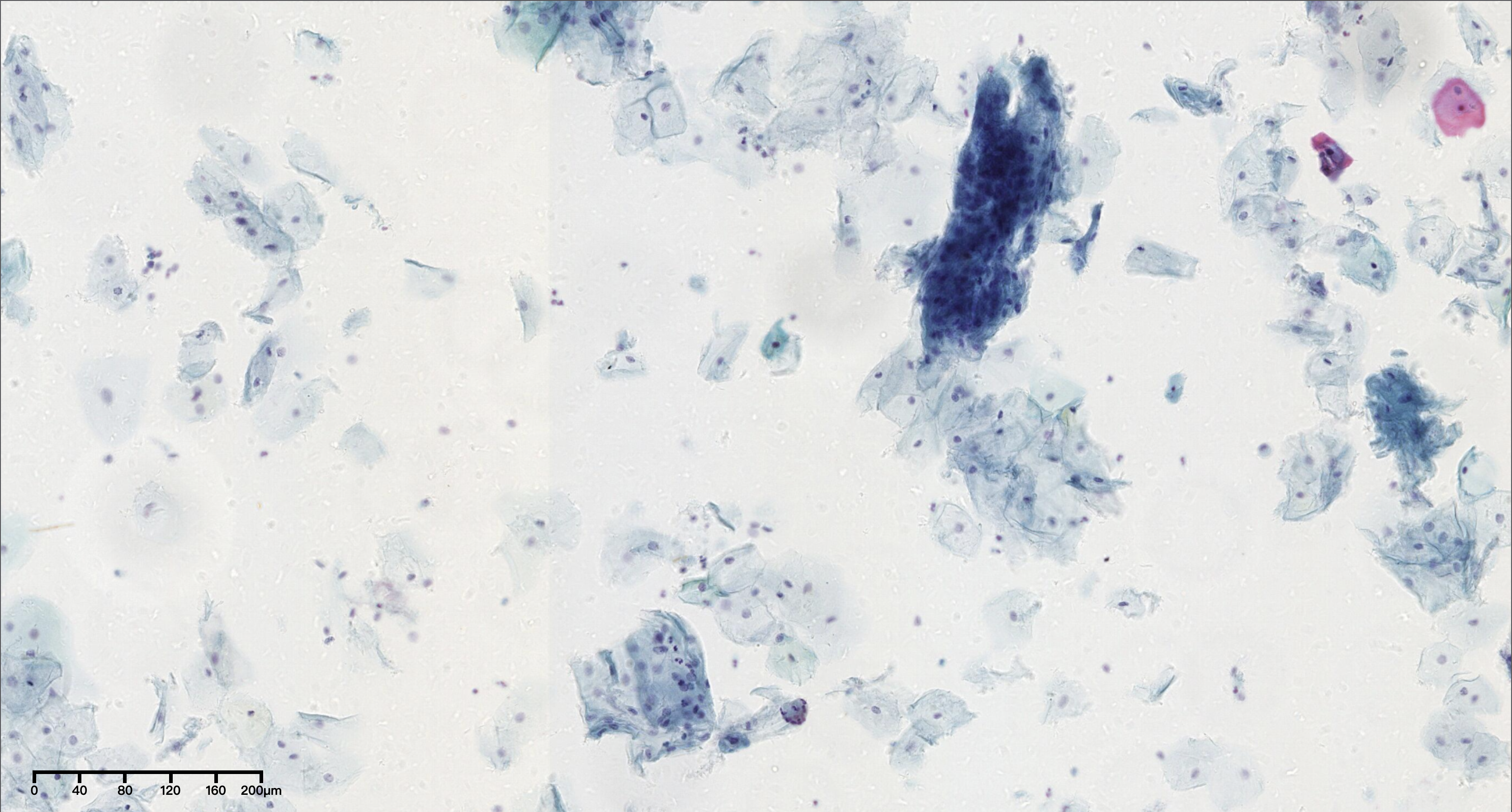

Supplement: Supplemental Information 24 — Staining images (LSIL, low-grade squamous intraepithelial lesion) [file peerj-13-20100-s024.png]

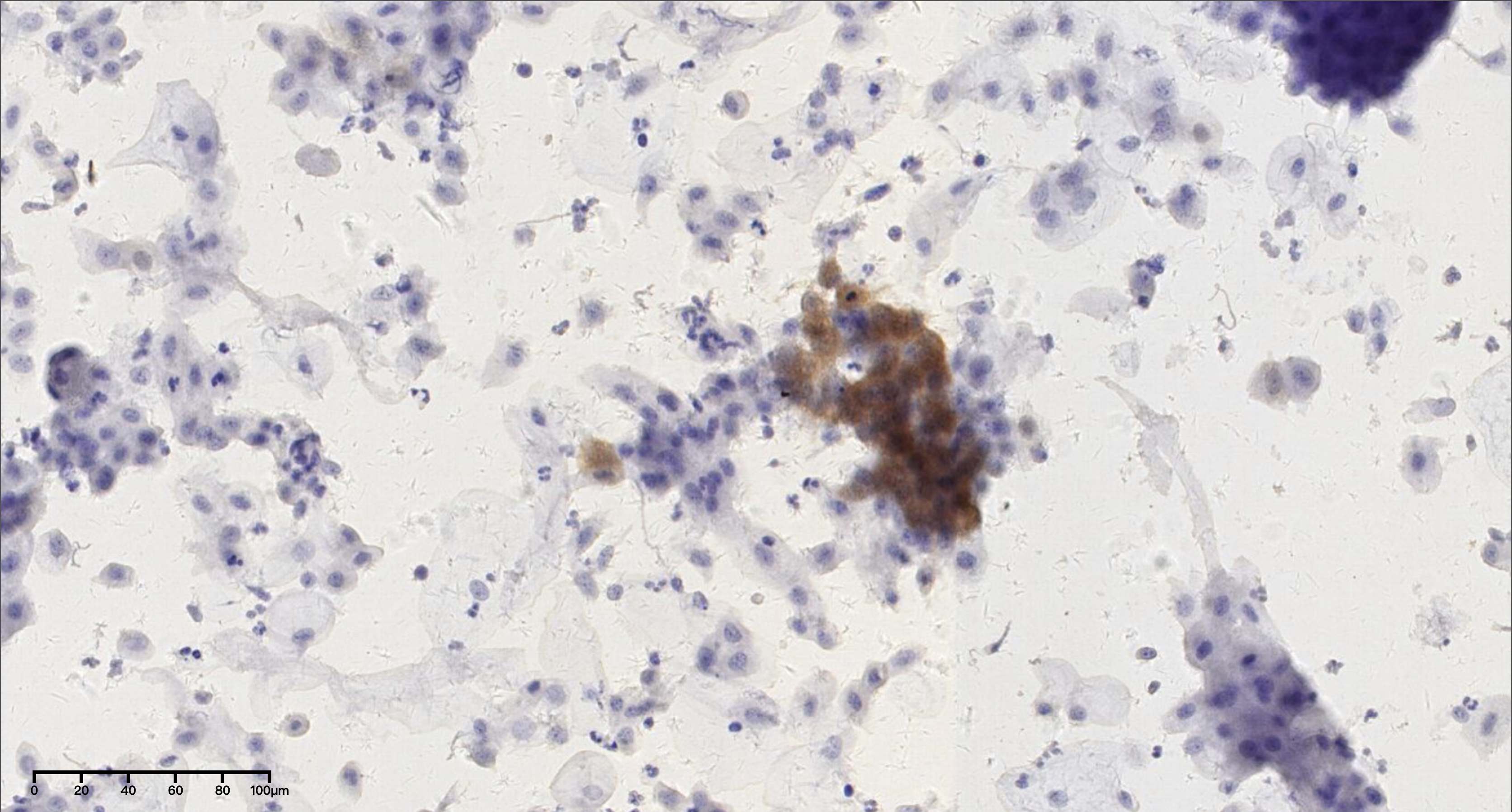

Supplement: Supplemental Information 25 — Brownish-yellow stained cervical epithelial cells that considered positive for p16. [file peerj-13-20100-s025.png]

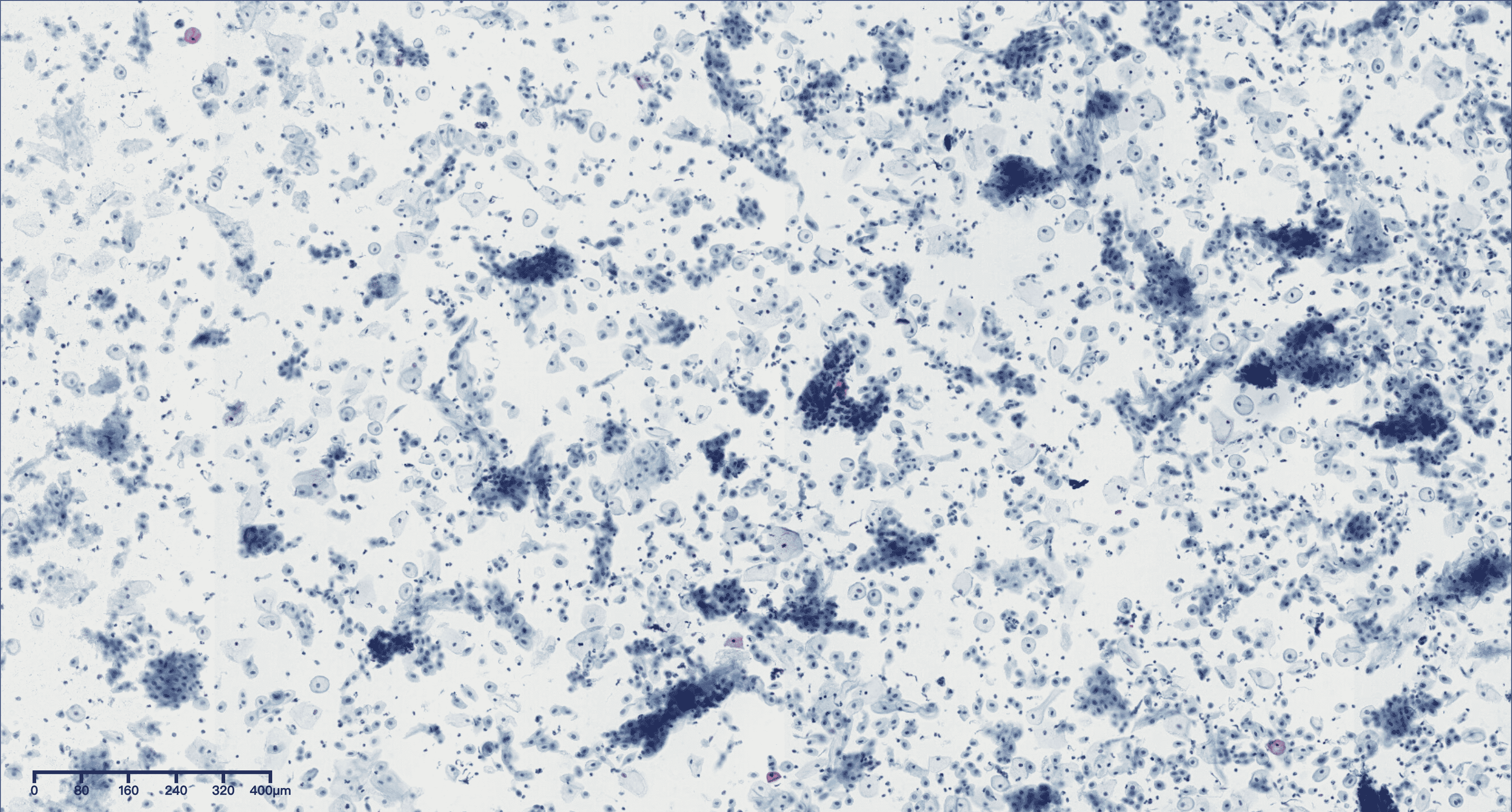

Supplement: Supplemental Information 26 — Staining images (ASC-H, atypical squamous cells cannot exclude high-grade lesion) [file peerj-13-20100-s026.png]

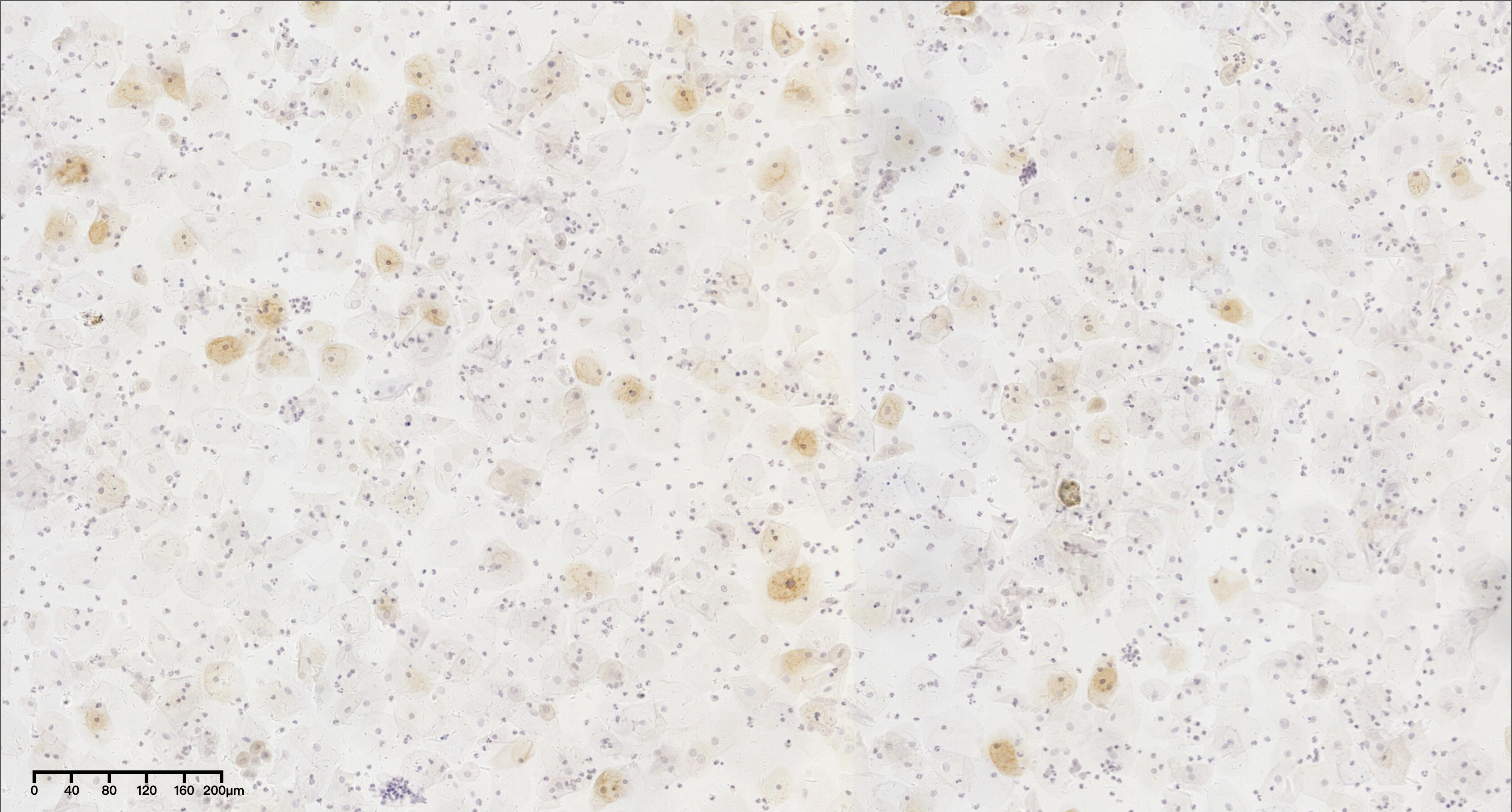

Supplement: Supplemental Information 27 — Brownish-yellow stained cervical epithelial cells that considered positive for p16. [file peerj-13-20100-s027.png]

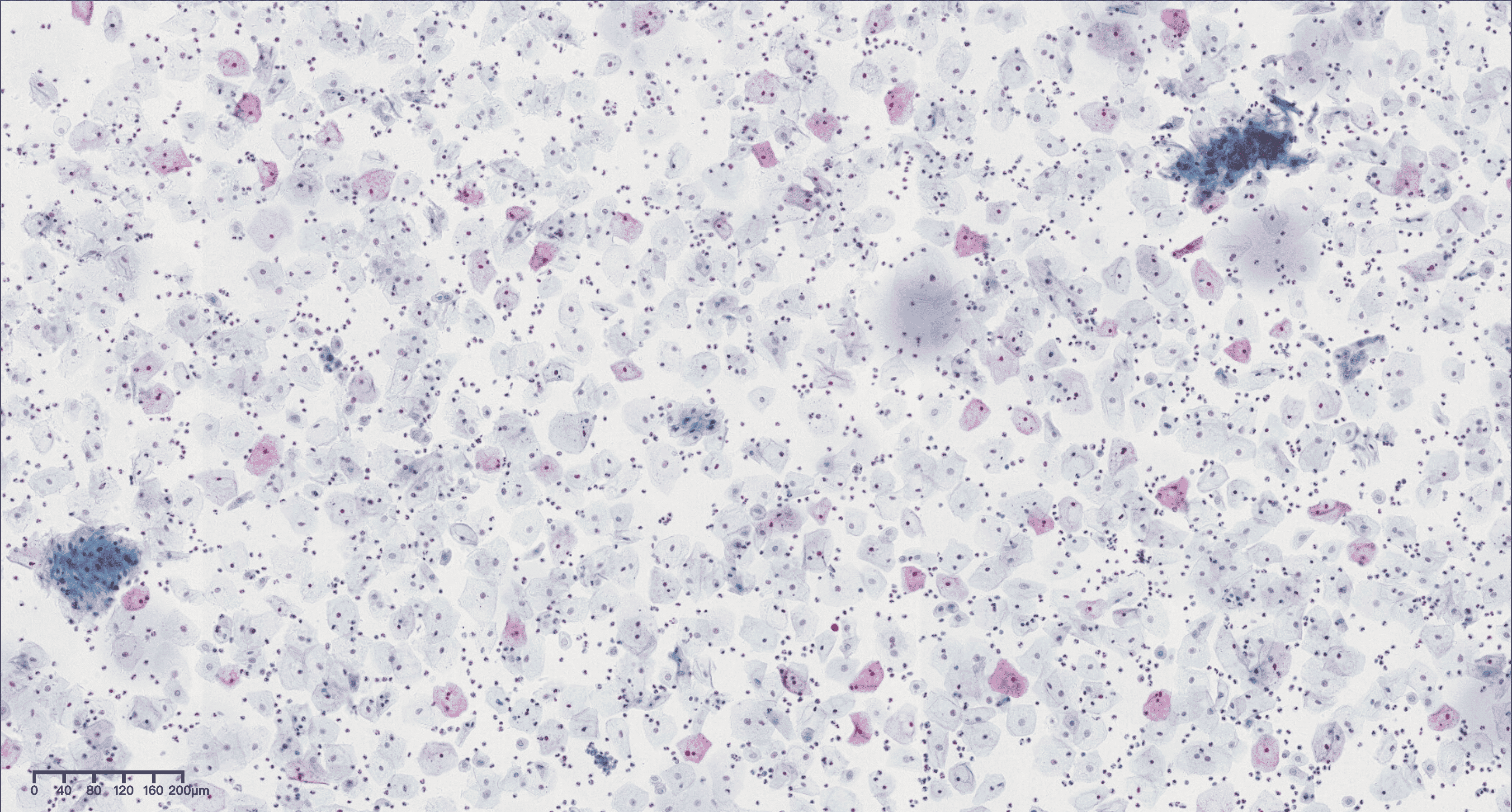

Supplement: Supplemental Information 28 — Staining images (LSIL, low-grade squamous intraepithelial lesion) [file peerj-13-20100-s028.png]

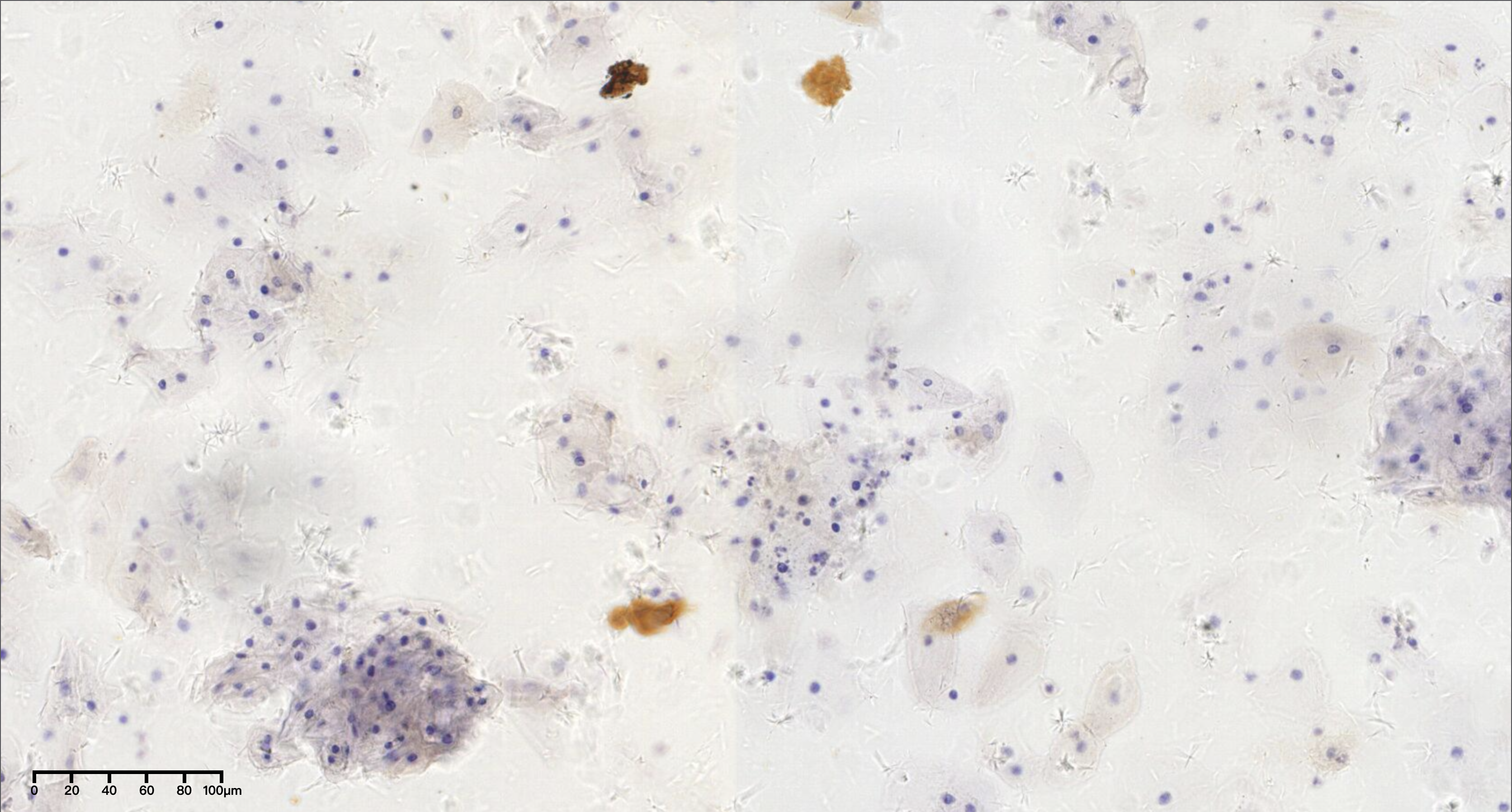

Supplement: Supplemental Information 29 — Brownish-yellow stained cervical epithelial cells that considered positive for p16. [file peerj-13-20100-s029.png]

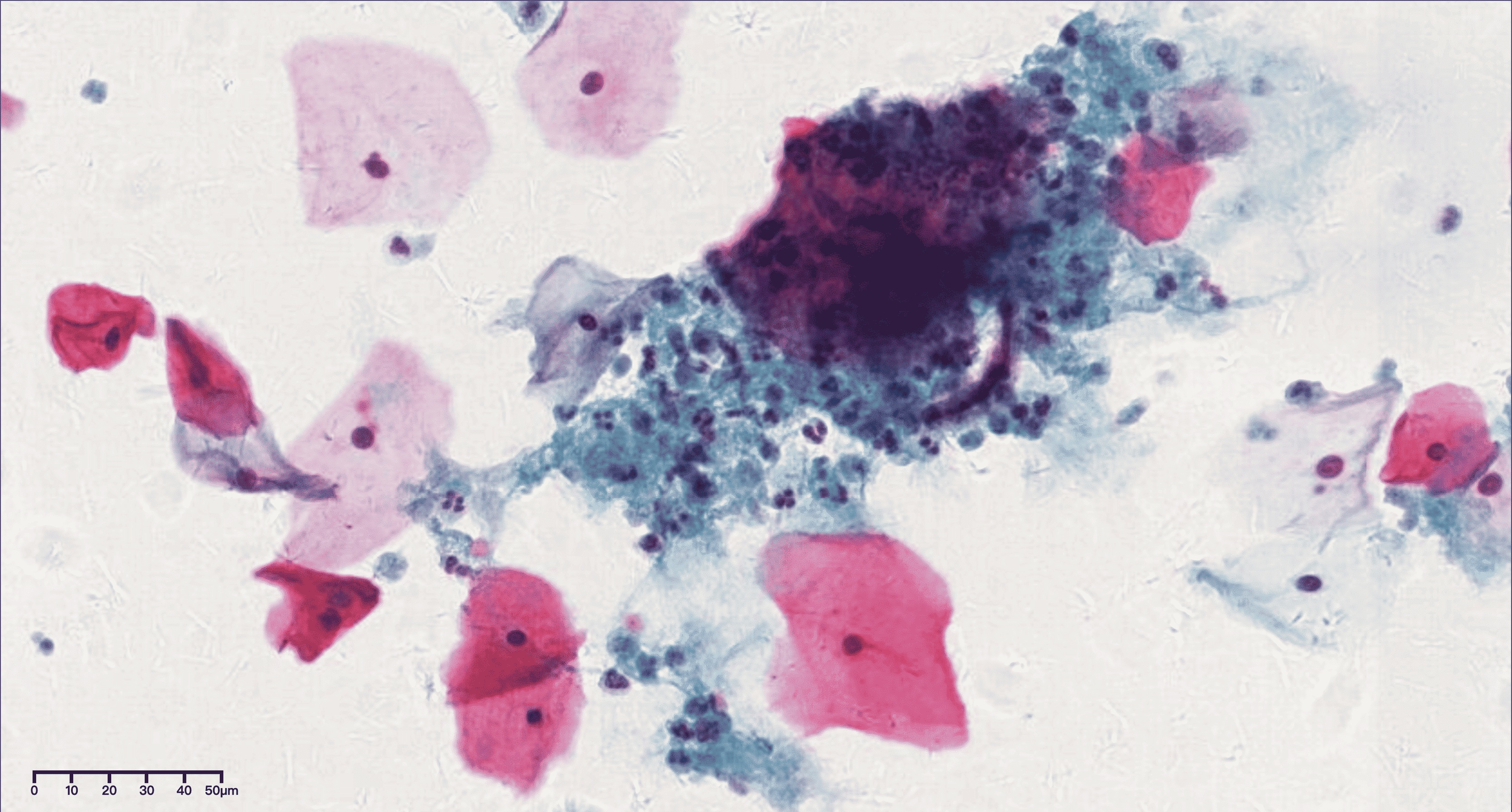

Supplement: Supplemental Information 30 — Staining images (LSIL, low-grade squamous intraepithelial lesion) [file peerj-13-20100-s030.png]

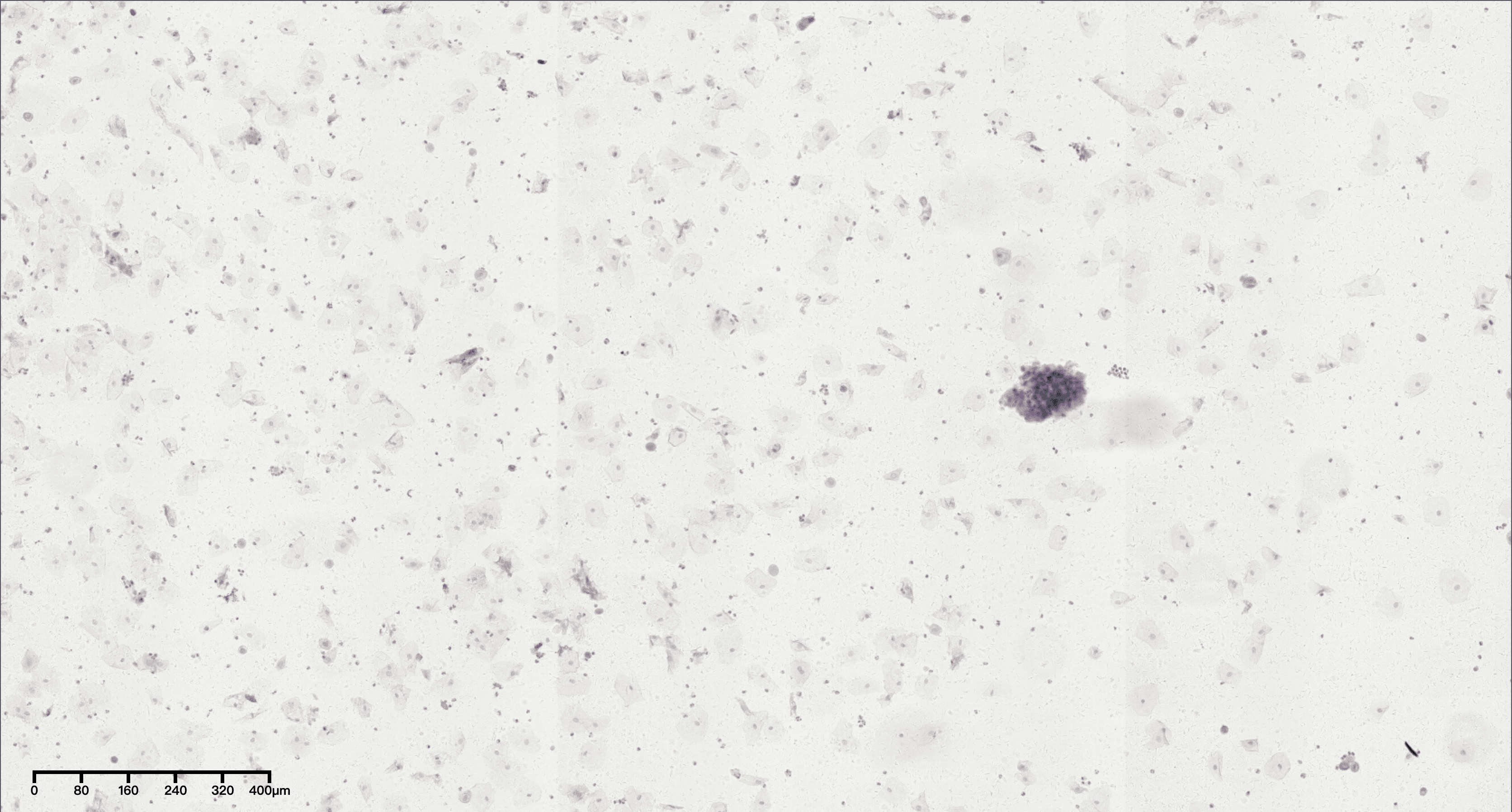

Supplement: Supplemental Information 31 — Brownish-yellow stained cervical epithelial cells that considered positive for p16. [file peerj-13-20100-s031.png]

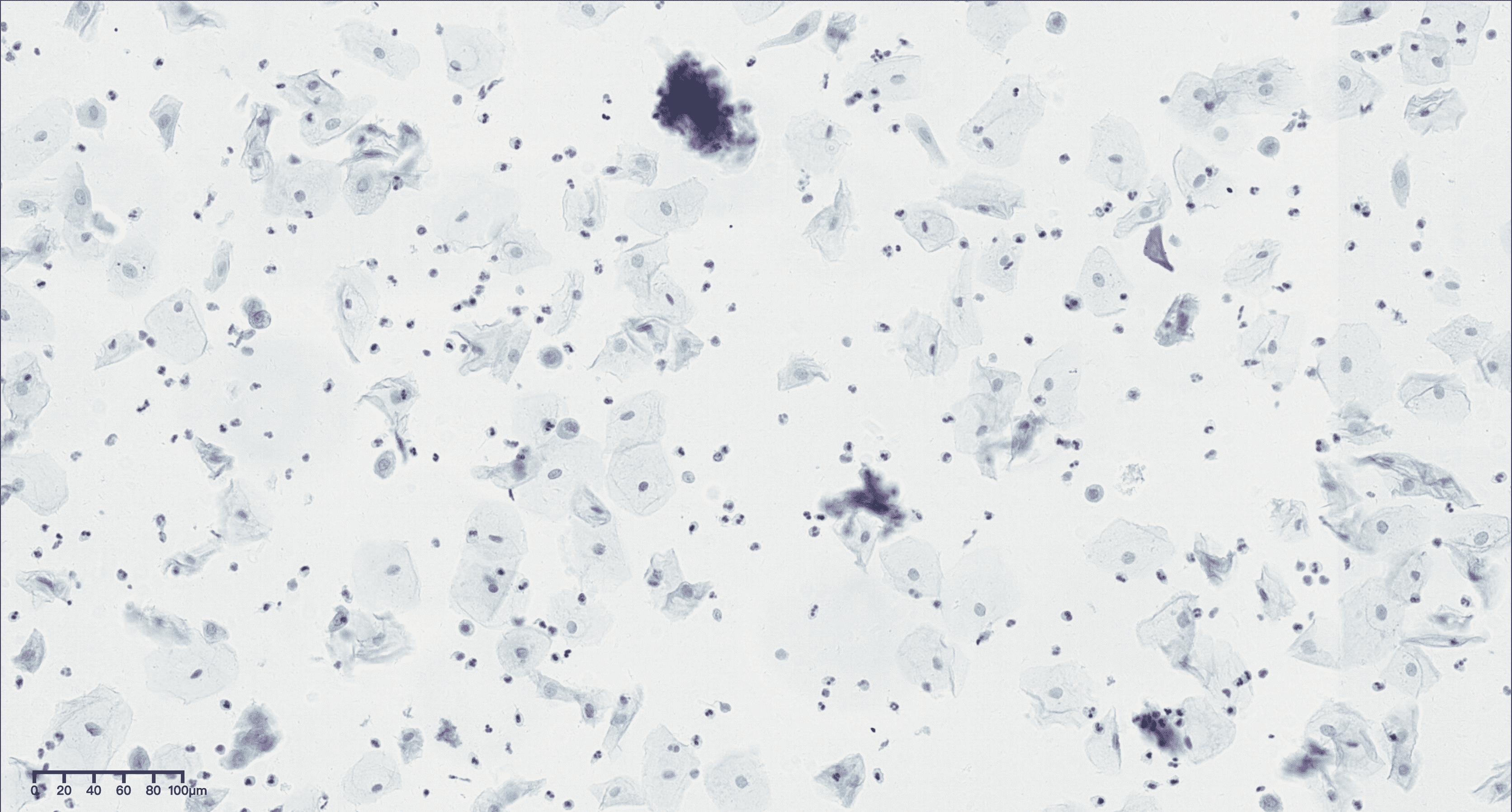

Supplement: Supplemental Information 32 — Staining images (LSIL, low-grade squamous intraepithelial lesion) [file peerj-13-20100-s032.png]

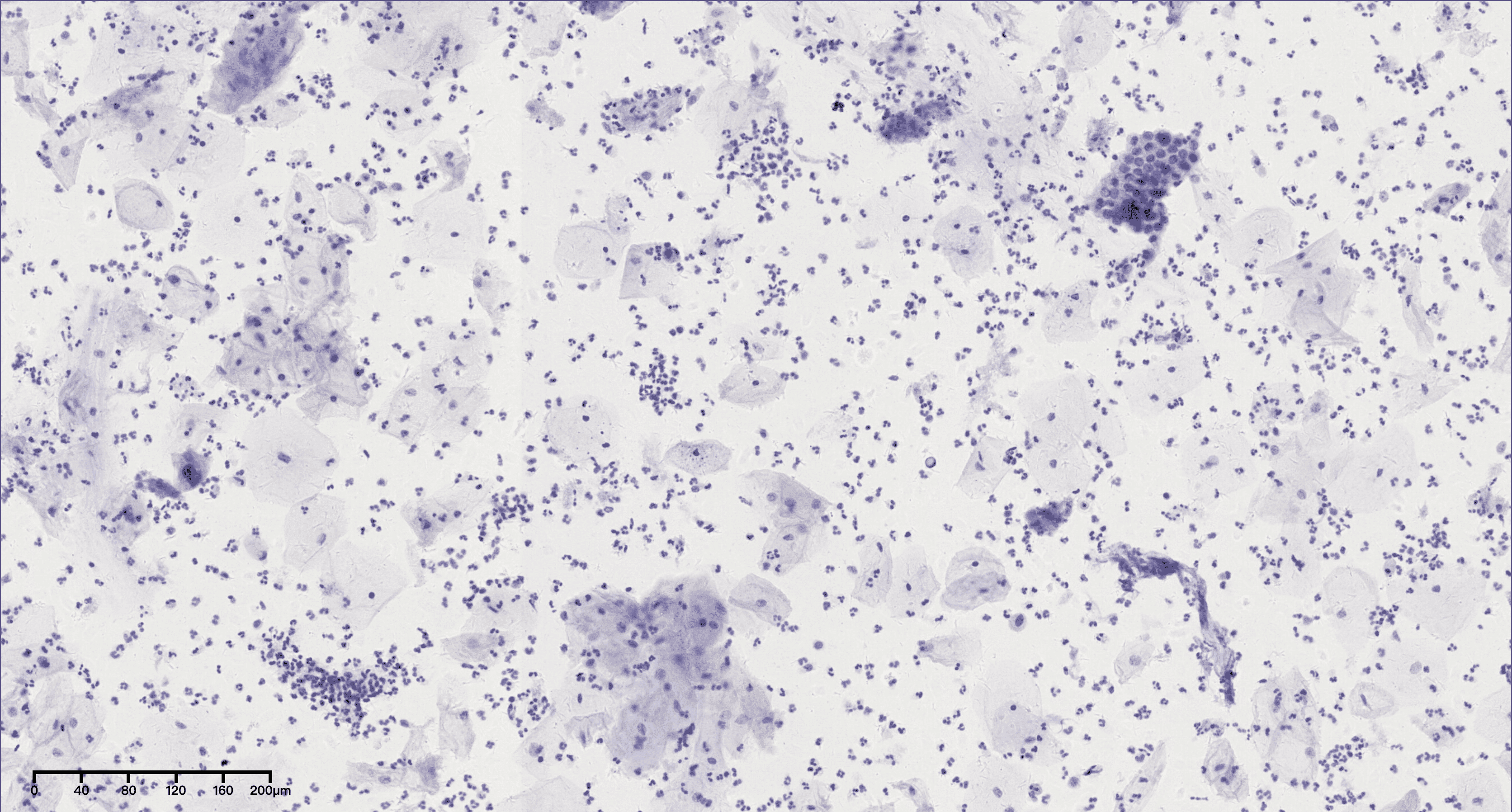

Supplement: Supplemental Information 33 — Brownish-yellow stained cervical epithelial cells that considered positive for p16. [file peerj-13-20100-s033.png]

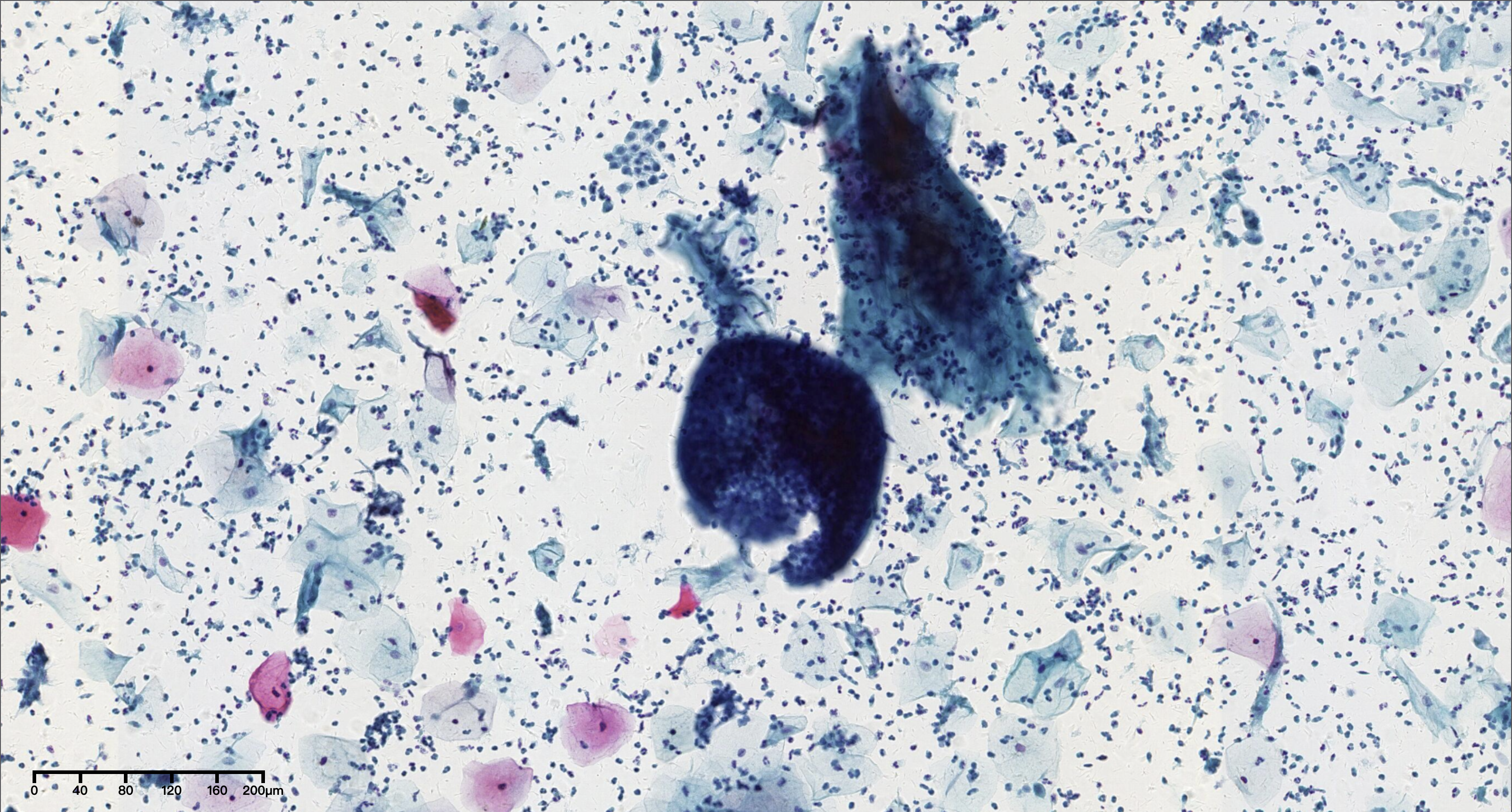

Supplement: Supplemental Information 34 — Staining images (LSIL, low-grade squamous intraepithelial lesion) [file peerj-13-20100-s034.png]

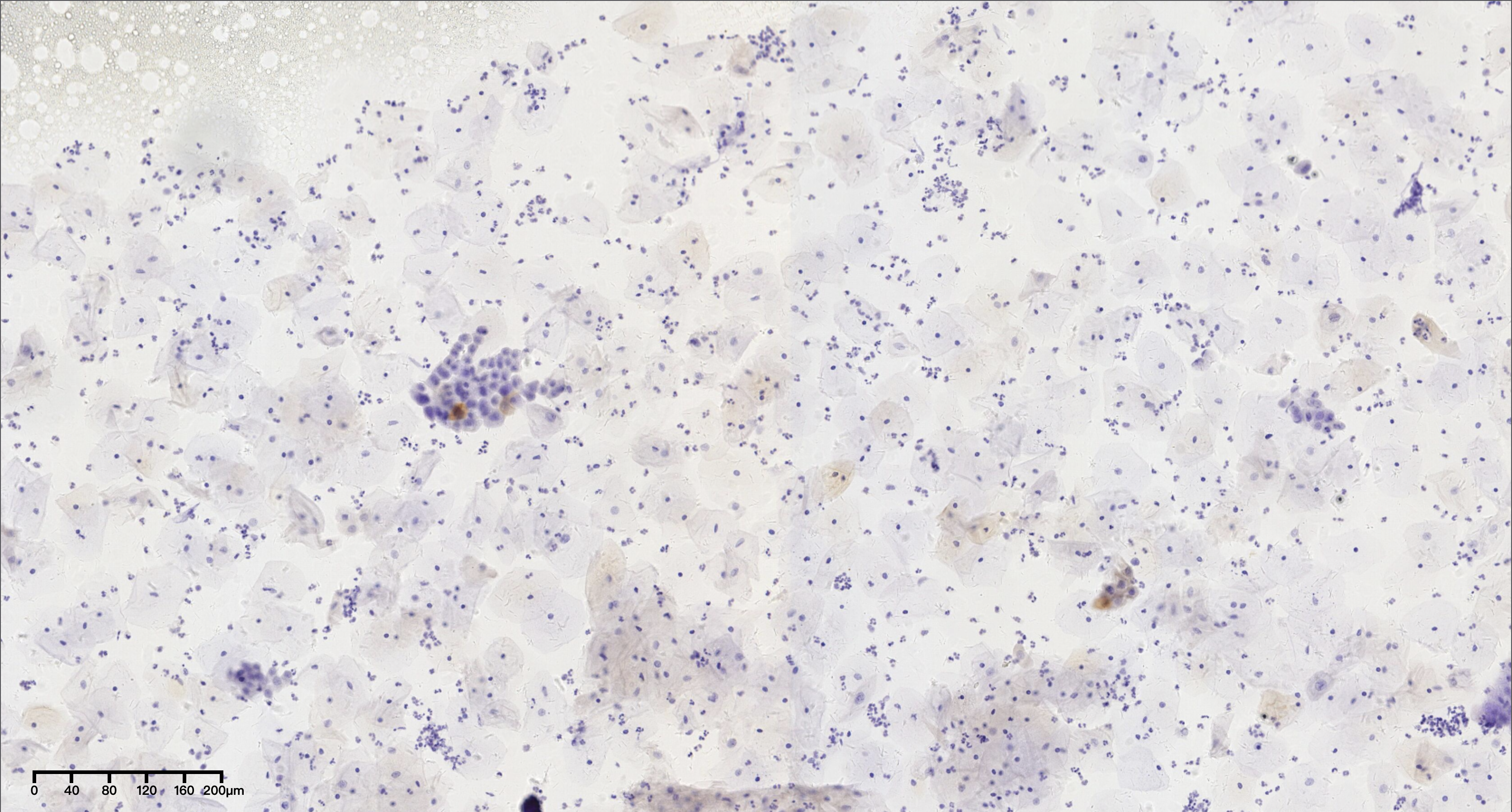

Supplement: Supplemental Information 35 — Brownish-yellow stained cervical epithelial cells that considered positive for p16. [file peerj-13-20100-s035.png]

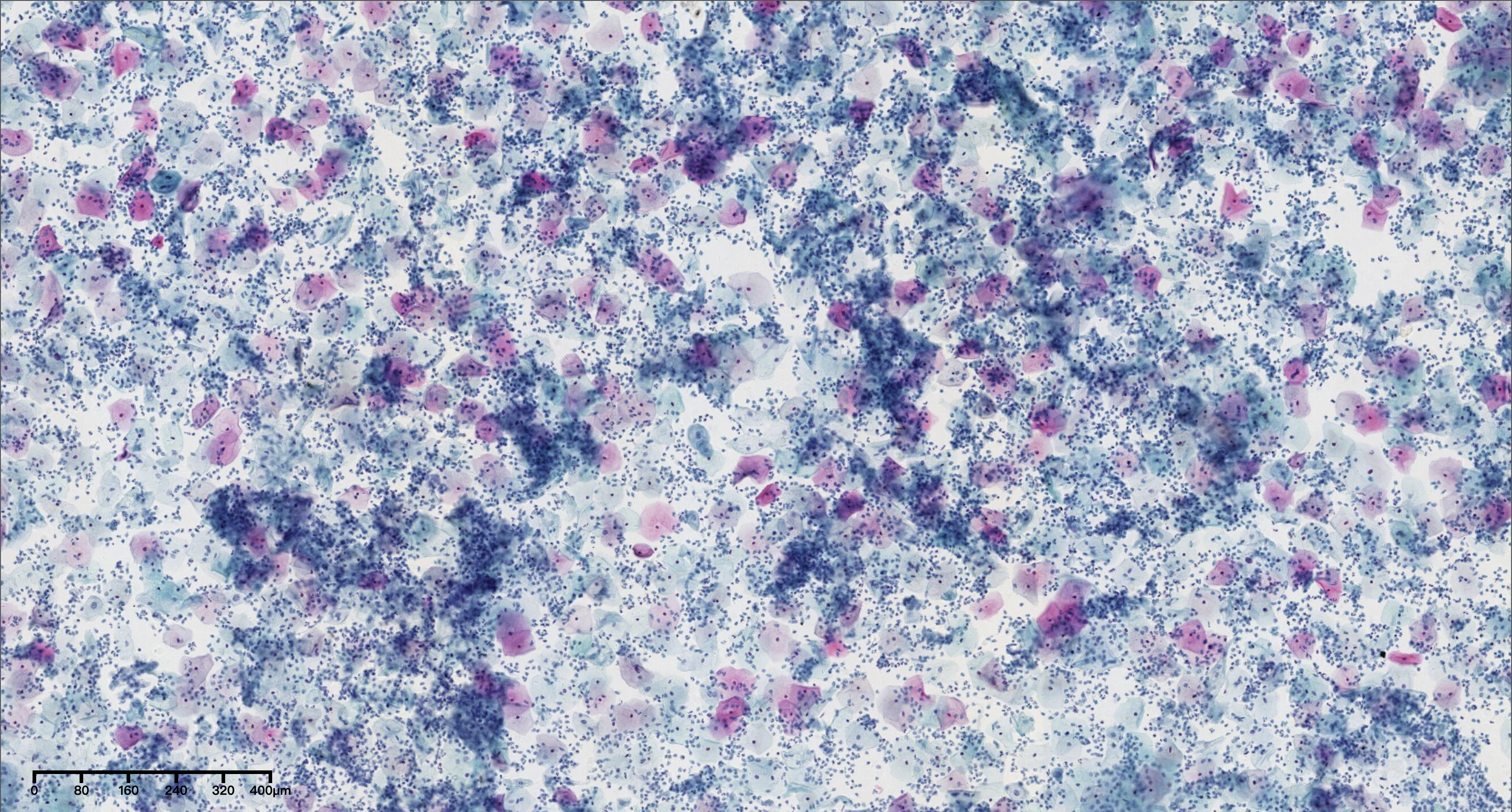

Supplement: Supplemental Information 36 — Staining images (LSIL, low-grade squamous intraepithelial lesion) [file peerj-13-20100-s036.png]

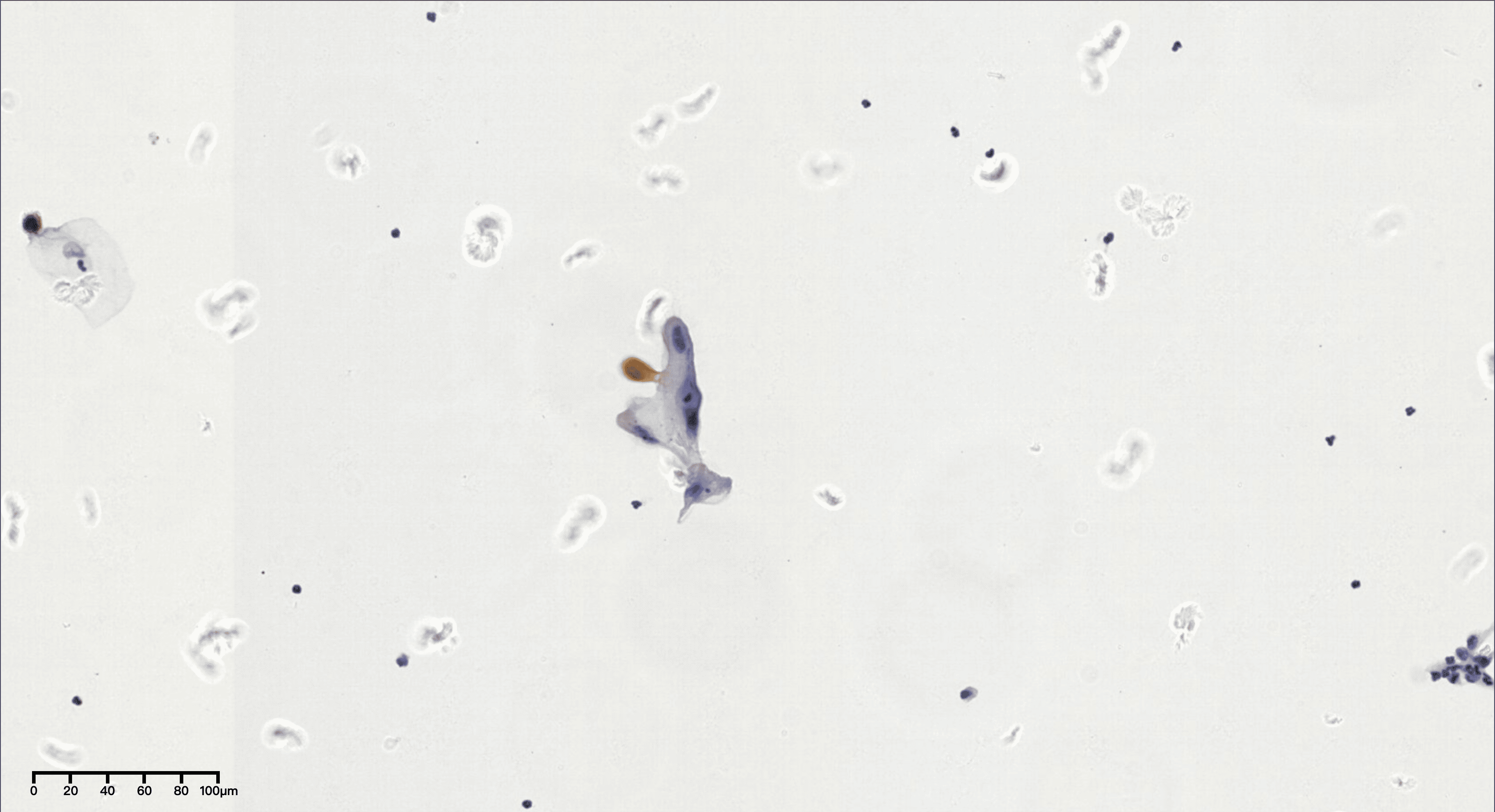

Supplement: Supplemental Information 37 — Brownish-yellow stained cervical epithelial cells that considered positive for p16. [file peerj-13-20100-s037.png]

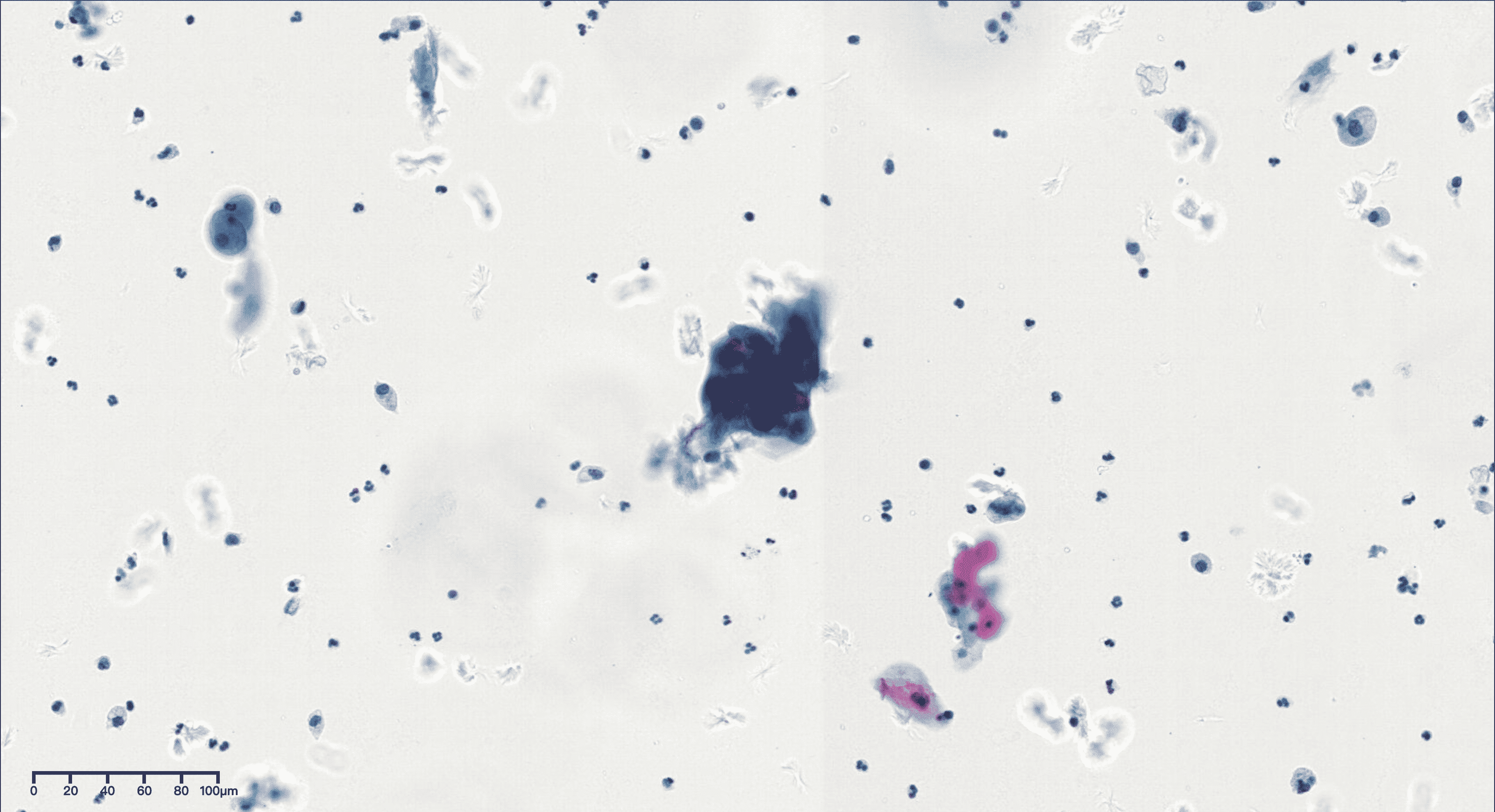

Supplement: Supplemental Information 38 — Staining images (LSIL, low-grade squamous intraepithelial lesion) [file peerj-13-20100-s038.png]

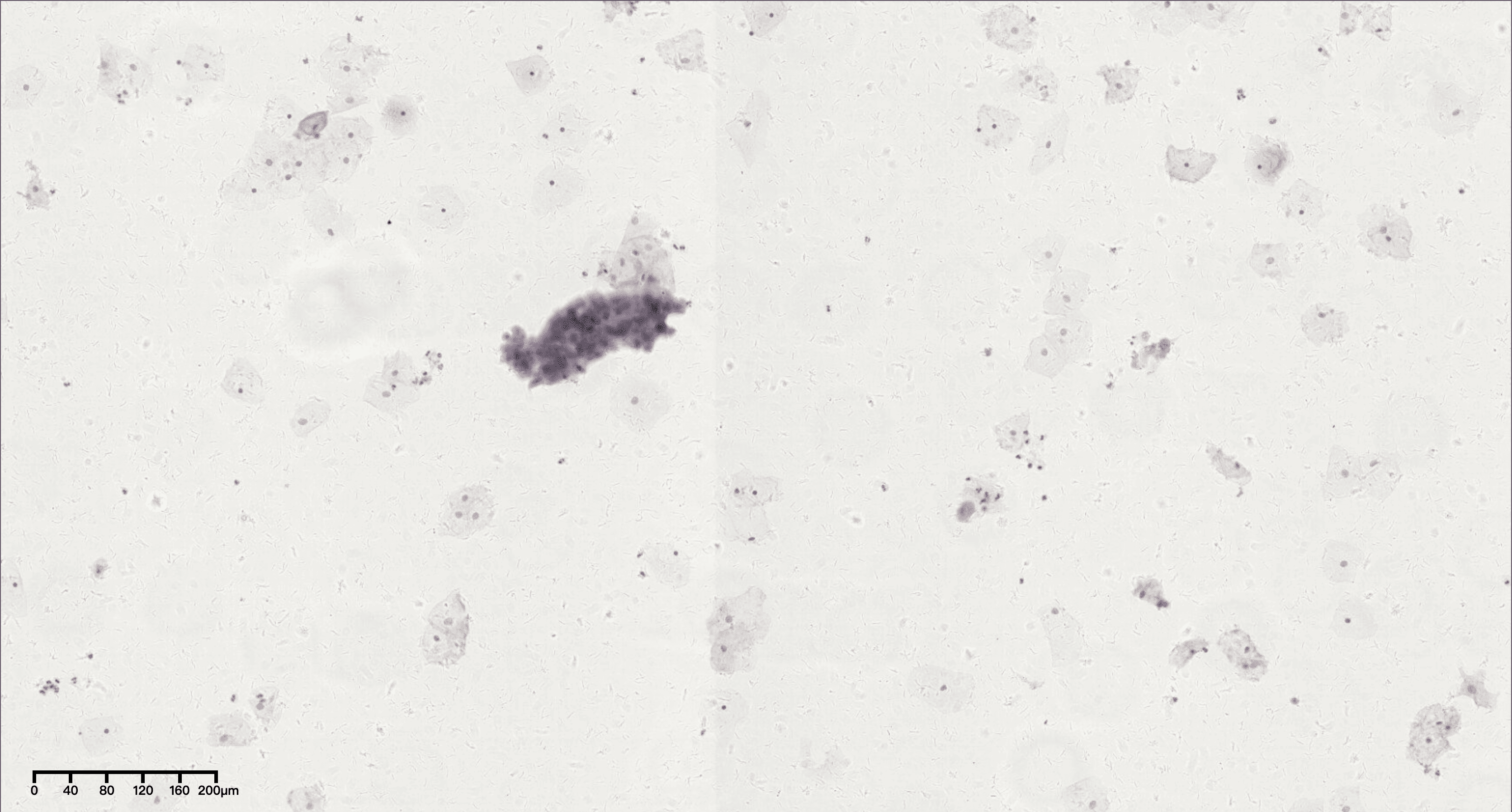

Supplement: Supplemental Information 39 — Brownish-yellow stained cervical epithelial cells that considered positive for p16. [file peerj-13-20100-s039.png]

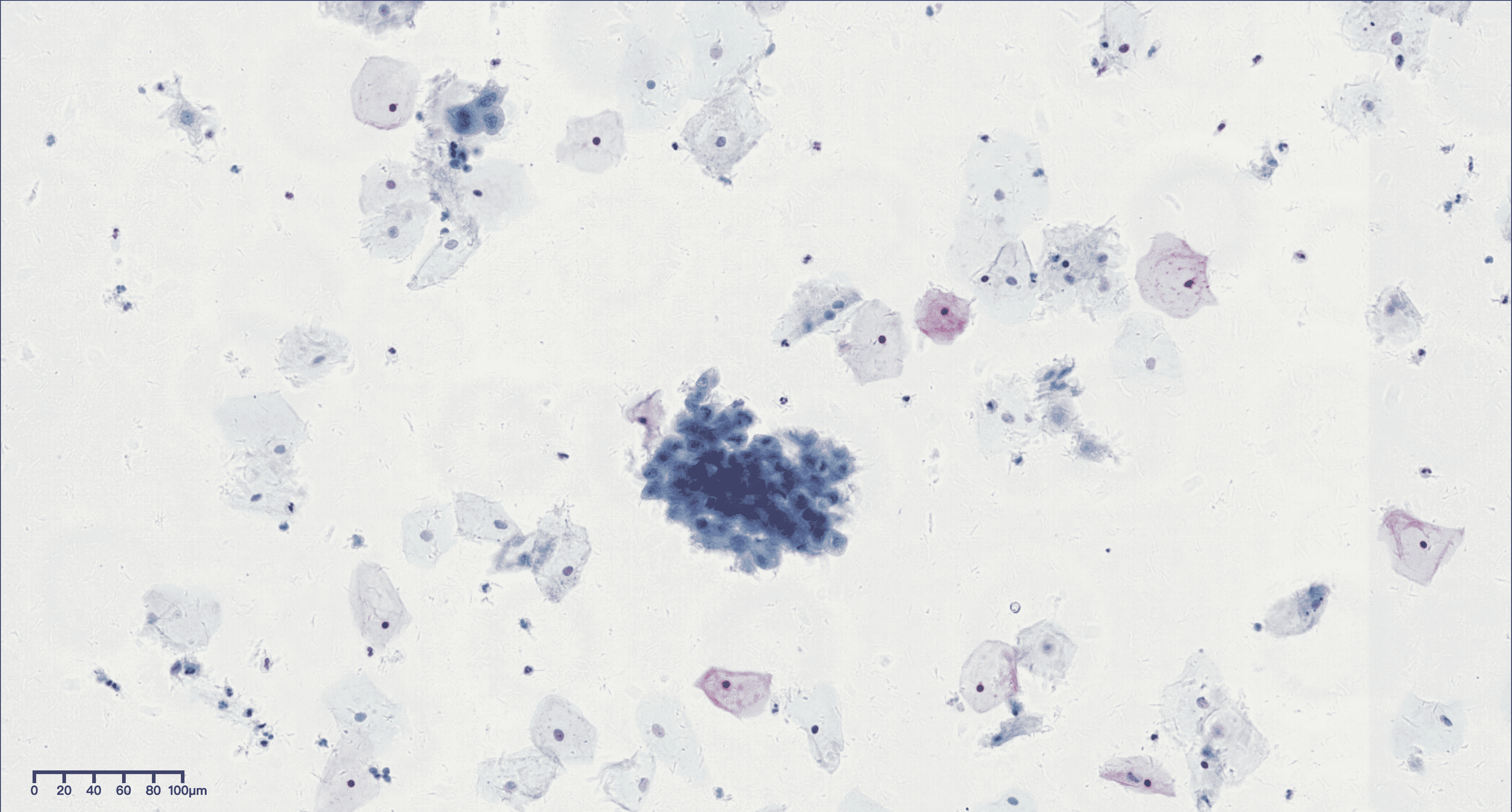

Supplement: Supplemental Information 40 — Staining images (ASC-H, atypical squamous cells cannot exclude high-grade lesion) [file peerj-13-20100-s040.png]
