# Supplementary material for: Barriers to cervical cancer prevention and triage strategies: a study of knowledge, attitudes, and p16/Ki-67 dual-staining utility among high-risk women in Tuoli and Fuyun counties, Xinjiang
Source: PeerJ. 2025 Oct 2;13:e20100. doi: 10.7717/peerj.20100 (PMC12497396; doi:10.7717/peerj.20100)
Supplement: Supplemental Information 41 [file peerj-13-20100-s041.docx]

Data-Introducing

“Table 1,2,3and Fig 2”

Marital：1=“Married”，2=“Single/divorced/widowed”

Educational：1=“Primary or lower”，2=“High school”，3=“College and higher”

Employment：1=“unemployed”，2 = "service sector workers (e.g., retail and hospitality professionals, such as mall salespersons, supermarket cashiers, restaurant staff, hotel receptionists, hairdressers, domestic helpers)"，3 = "public servants (government and state employees, including military personnel, civil servants, public school teachers, public hospital doctors, researchers in public institutions)"，4 = "general laborers (including agricultural and factory workers, such as crop farmers, livestock breeders, factory assembly-line workers, construction laborers)"

Incomes：1=“≤30000”，2=“30000-60000”，3=“60000-100000”，4=“≥100000”

Awareness：1=“Awareness” ，0=“Unawareness”

Screening：1=“Ever screened” ，0=“Never screened”

“Fig 3”

2.5：1=“Once per year”，2=“Every three years”，3=“Every five years”，4=“Based on screening results”，5=“Follow medical advice”，6=“Unwilling”，7=“Others”

2.6：1=“Don't know”，2=“1 day”，3=“Within one week”，4=“In one month”，5=“Both, at doctor's discretion”，6=“Others”

2.8：1=“Mobile phone SMS”，2=“Telephone call”，3=“Report card”，4=“Ask the doctor yourself”，5=“Others”

2.9：1=“willing”，2=“unwilling”，3=“Uncertain”

2.11：1=“Lack of money for treatment”，2=“Asymptomatic, no treatment required”，3=“no time”，4=“Others”

2.12：1=“Yes”，2=“No”

2.13：1=“Yes”，2=“No”

“Fig 4”

2.3：1=“Required by physician/Doctor”，2=“Recommended by friend/family”，3=“Slogans and posters”，4=“Social welfare campaigns”，5=“Media”，6=“Others”

“Fig 5”

2.7：1=“More hygiene promotion and education”，2=“Take your own cervical sample”，3=“Parent organization, required to attend”，4=“Reduce the number of inspections and solve traffic problems”，5=“Persuasion by family or friends”，6=“Others”

“Fig 6”

2.10：1=“Too frequent inspections”，2=“Long way to go, too much hassle”，3=“Screening too hard to go through again”，4=“Feeling healthy/results not serious enough to warrant a review”，5=“Prefer to be re-tested at county and city hospitals”，6=“Too busy to have time”，7=“Others”
