# Supplementary material for: Barriers to cervical cancer prevention and triage strategies: a study of knowledge, attitudes, and p16/Ki-67 dual-staining utility among high-risk women in Tuoli and Fuyun counties, Xinjiang
Source: PeerJ. 2025 Oct 2;13:e20100. doi: 10.7717/peerj.20100 (PMC12497396; doi:10.7717/peerj.20100)
Supplement: Supplemental Information 46 [file peerj-13-20100-s046.doc]

# 宫颈癌筛查调查表

填表说明：请在选项前的“□”内打人，除标明“多选”外，均为单选。

第一部分：基本信息

**1.1 ID: __________**

**1.2民族：**

£1=汉族

£2=蒙族

£3=其他

**1.3 婚姻状况：**

£1=未婚

£2=已婚

£3=离异

£4=丧偶

£5=其他，请注明**: __________**

**1.4 文化程度：**

£1=未受过教育

£2=小学

£3=初中

£4=高中（含中专、技校）

£5=本科及以上（含大专）

**1.5 职业：**

£1=无业

£2=农民

£3=军人

£3=上级组织，要求参加

£4=企业、商业、服务业人员

£5=国家机关、党群组织、事业单位人员

£6=工人

£7=其他，请注明**: __________**

**1.6 去年您的全家总收入（元）：**

£1=3万以下

£2=3-6万（不含6万）

£3=6-10万（不含10万）

£4=10万以上（含10万）

**1.7 您的医疗支付方式是：（可多选）**

£1=城镇职工医疗保险

£2=城镇居民医疗保险

£3=新农村合作医疗

£4=商业医疗保险

£5=贫困救助

£6=全自费

£7=其他，请注明**: __________**

第二部分：对宫颈癌筛查的认知和接受程度

**2.1 您是否听说过人乳头瘤病毒（HPV）？**

£1=是

£2=否

**2.2 您是否听说过宫颈癌筛查？**

£1=是

£2=否（跳至2.5）

**2.3 您从哪里获得宫颈癌筛查的信息？（多选）**

£1=村医生或上级（医院）通知

£2=家人/朋友介绍

£3=标语和宣传画

£4=社会公益宣传

£5=媒体（广播/电视/报纸/杂志/网络）

£6=其他，请注明**: __________**

**2.4 您听说过的宫颈癌筛查方法有哪些？（可多选）**

£1=不知道

£2=细胞学检查（巴氏涂片、液基细胞学）

£3=醋酸/碘液肉眼观察（VIA/VILI）

£4=HPV 检测（如HC2、careHPV、Cobas4800）

£5=阴道镜检查

£6=其他，请注明**: __________**

**2.5 您愿意多久进行一次宫颈癌检查？**

£1=每一年一次

£2=每三年一次

£3=每五年一次

£4=根据检查结果制定

£5=遵医嘱

£6=不愿意

£7=其他，请注明**: __________**

**2.6 您可以接受的最长的检查结果报告时间？**

£1=不知道

£2=当天

£3=一周内

£4=半月内

£5=一月内

£6=均可，由医生决定

£7=其他，请注明**: __________**

**2.7 您认为怎样能让不愿参加筛查的人参加（多选）：**

£1=多做卫生宣传教育

£2=自己取宫颈样本

£3=上级组织，要求参加

£4=减少检查次数，解决交通问题

£5=家人或朋友劝说

£6=其他，请注明**: __________**

**2.8 您希望本次筛查结果以何种方式反馈？**

£1=手机短信

£2=电话通知

£3=报告单

£4=自己问医生

£5=其他，请注明**: __________**

**2.9 若医生通知复查，您愿意参加吗？**

£1=愿意（跳转2.11）

£2=不愿意

£3=不确定（跳转2.11）

**2.10** **哪些原因可能使您不愿意复查？（多选）**

£1=检查次数太频繁

£2=路途遥远，太麻烦

£3=筛查太难受，不愿意再经历

£5=感觉自己身体健康/结果不严重，没必要复查

£6=更愿意到县、市级医院重新检查

£7=太忙，没时间

£8=其他，请注明**: __________**

**2.11 在何种情况下，您可能不愿意进行治疗：**

£1=治疗太贵

£2-无症状，不需治疗

£3=没时间

£4=其他，请注明**: __________**

**2.12 您是否听说过预防性HPV/宫颈癌疫苗？**

£1=是

£2=否

**2.13 您是否愿意让您的孩子接种HPV疫苗？**

£1=是

£2=否
