# Supplementary material for: Barriers to cervical cancer prevention and triage strategies: a study of knowledge, attitudes, and p16/Ki-67 dual-staining utility among high-risk women in Tuoli and Fuyun counties, Xinjiang
Source: PeerJ. 2025 Oct 2;13:e20100. doi: 10.7717/peerj.20100 (PMC12497396; doi:10.7717/peerj.20100)
Supplement: Supplemental Information 47 [file peerj-13-20100-s047.doc]

# Cervical Cancer Screening Questionnaire

Instructions for completing the form: Please tick the "£" before the options, which are all single choice, except for those marked "multiple choice".

Part I: Basic Information

**1.1 ID: __________**

**1.2 Ethnicity:**

□ 1 = Han

□ 2 = Mong

□ 3 = Other

**1.3 Marital status:**

□ 1 = Unmarried

□ 2 = Married

□ 3 = Divorced

□ 4 = Widowed

□ 5 = Other, please specify: __________

**1.4 Educational attainment**

□ 1 = uneducated

□ 2 = Primary

□ 3 = Junior High School

□ 4 = High school (including secondary and technical schools)

□ 5 = Bachelor's degree and above (including tertiary)

**1.5 Employment status**

□ 1 = no work

□ 2 = Farmers

□ 3 = Military

□ 3 = parent organization, required to attend Population

£4 = Enterprises, business, service workers

□ 5 = State organs, party organizations and institutions

□ 6 = Workers

□ 7 = Other, please specify: __________

**1.6 Your total family income last year (RMB):**

□ 1 = less than 30,000

□ 2 = 30-60,000 (excluding 60,000)

□ 3 = 60,000-100,000 (excluding 100,000)

□ 4 = 100,000 or more (inclusive)

**1.7 How do you pay for your health care: (multiple choice)**

□ 1 = urban workers' health insurance

□ 2 = urban residents' health insurance

□ 3 = New Rural Co-operative Medical Care

□ 4 = Commercial medical insurance

□ 5 = Poverty assistance

□ 6 = fully self-funded

□ 7 = Other, please specify: __________

Part II: Awareness and acceptance of cervical cancer screening

**2.1 Have you heard of human papillomavirus (HPV)?**

□ 1 = Yes

£2=No

**2.2 Have you heard of cervical cancer screening?**

□ 1 = Yes

□ 2 = No (skip to 2.5)

**2.3 Where did you get information about cervical cancer screening? (Multiple choice)**

□ 1 = village doctor or higher level (hospital) notification

□ 2 = family/friend referral

□ 3 = Slogans and posters

□ 4 = Social welfare campaigns

□ 5 = Media (radio/television/newspapers/magazines/web)

□ 6 = Other, please specify: __________

**2.4 What are some of the cervical cancer screening methods you have heard of? (Multiple answers allowed)**

□ 1 = Don't know

□ 2 = cytological examination (Pap smear, liquid-based cytology)

□ 3 = Visual inspection with acetic acid/iodine solution (VIA/VILI)

□ 4 = HPV detection (e.g., HC2, careHPV, Cobas4800)

□ 5 = Colposcopy

□ 6 = Other, please specify: __________

**2.5** **How often would you be willing to be screened for cervical cancer?**

□ 1 = once per year

□ 2 = every three years

□ 3 = every five years

□ 4 = based on screening results

□ 5 = follow medical advice

□ 6 = unwilling

□ 7 = Other, please specify: __________

**2.6 What is the longest acceptable time to get your results reported?**

□ 1 = Don't know

□ 2 = 1 day

□ 3 = within one week

□ 4 = within half a month

□ 5 = In one month

□ 6 = both, at doctor's discretion

□ 7 = Other, please specify: __________

**2.7** **What do you think would make it possible for people who do not want to take part in screening to do so (multiple choice):**

□ 1 = More hygiene promotion and education

□ 2 = Take your own cervical sample

□ 3 = parent organization, required to attend

□ 4 = Reduce the number of inspections and solve traffic problems

□ 5 = Persuasion by family or friends

□ 6 = Other, please specify: __________

**2.8 How would you like to have feedback on the results of this screening?**

□ 1 = mobile phone SMS

□ 2 = telephone call

□ 3 = report card

□ 4 = Ask the doctor yourself

□ 5 = Other, please specify: __________

**2.9 Would you be willing to have a second test if your doctor called?**

□ 1 = willing (skip 2.11)

□ 2 = unwilling

□ 3 = Uncertain (skip 2.11)

**2.10 What keeps you from having a second test? (Multiple choice)**

□ 1 = Too frequent inspections

□ 2 = Long way to go, too much hassle

□ 3 = Screening too hard to go through again

□ 5 = Feeling healthy/results not serious enough to warrant a review

□ 6 = Prefer to be re- tested at county and city hospitals

□ 7 = Too busy to have time

□ 8 = Other, please specify: __________

**2.11** **Circumstances in which you may not wish to have treatment:**

□ 1 = Lack of money for treatment

□ 2 =asymptomatic, no treatment required

□ 3 = no time

□ 4 = Other, please specify: __________

**2.12 Have you heard about the HPV/cervical cancer vaccine?**

□ 1 = Yes

£2=No

**2.13 Would you like your child to be vaccinated against HPV?**

□ 1 = Yes

£2=No
